# Value and limitations of machine learning in high-frequency nutrient data for gap-filling, forecasting, and transport process interpretation

**DOI:** 10.1007/s10661-023-11519-9

**Published:** 2023-06-27

**Authors:** Victoria Barcala, Joachim Rozemeijer, Kevin Ouwerkerk, Laurens Gerner, Leonard Osté

**Affiliations:** 1 Unit Inland Water Systems, Daltonlaan 600, 3584 BK Utrecht, The Netherlands; 2 Unit Subsurface and Groundwater Systems, Daltonlaan 600, 3584 BK Utrecht, The Netherlands; 3 Water Board Rijn and IJssel, Liemersweg 2, 7006 GG Doetinchem, The Netherlands

**Keywords:** Water management, Missing data, Data-based models, Random forest, Groundwater surface water interactions

## Abstract

High-frequency monitoring of water quality in catchments brings along the challenge of post-processing large amounts of data. Moreover, monitoring stations are often remote and technical issues resulting in data gaps are common. Machine learning algorithms can be applied to fill these gaps, and to a certain extent, for predictions and interpretation. The objectives of this study were (1) to evaluate six different machine learning models for gap-filling in a high-frequency nitrate and total phosphorus concentration time series, (2) to showcase the potential added value (and limitations) of machine learning to interpret underlying processes, and (3) to study the limits of machine learning algorithms for predictions outside the training period. We used a 4-year high-frequency dataset from a ditch draining one intensive dairy farm in the east of The Netherlands. Continuous time series of precipitation, evapotranspiration, groundwater levels, discharge, turbidity, and nitrate or total phosphorus were used as predictors for total phosphorus and nitrate concentrations respectively. Our results showed that the random forest algorithm had the best performance to fill in data-gaps, with *R*^2^ higher than 0.92 and short computation times. The feature importance helped understanding the changes in transport processes linked to water conservation measures and rain variability. Applying the machine learning model outside the training period resulted in a low performance, largely due to system changes (manure surplus and water conservation) which were not included as predictors. This study offers a valuable and novel example of how to use and interpret machine learning models for post-processing high-frequency water quality data.

**Supplementary Information:**

The online version contains supplementary material available at 10.1007/s10661-023-11519-9.

## Introduction

Intensive agriculture is an important source of nutrients in surface waters (Bol et al., 2018; Van der Grift et al., 2016; Van der Salm et al., 2012; Withers et al., 2014). High total phosphorus (TP) or high nitrate (NO_3_) concentrations are two of the parameters that can lead to a poor ecological quality status in surface waters. High NO_3_ and TP concentrations are being pointed out as one of the main causes of biodiversity loss (Dise et al., 2011; Porter et al., 2013) and algae blooms (Withers & Haygarth, 2007). In many cases, the nutrients lost to surface water do not just originate from the freshly applied manure or fertilizer, but from the nutrient legacy accumulated in the soil which is transported into surface waters through natural of artificial drainage systems (Bieroza et al., 2019; Lucas et al., 2021; Sharpley et al., 2013). Realistic goals and appropriate mitigation measures are needed (Schoumans et al., 2014). Therefore, it is central for the water authorities to monitor the water quality of surface waters and quantify the effect that nutrient sources and system changes have in the transport processes and leaching of nutrients into larger water systems.

High-frequency monitoring of water quality data offers a detailed understanding of the processes involved in nutrient transport (Rode et al., 2016; Rozemeijer et al., 2010a, b). However, technicians often face the challenge to deal with large data volumes and missing data (Zhang et al., 2019). It is often the case that sensors and autoanalyzers have technical problems which can result in a significant number of gaps in the data. The post processing of the data, including identification of errors and filling missing values can be time consuming and the final result depends on the individual who does the post processing (Jones et al., 2021). Furthermore, the amount of data collected by high-frequency sensors can easily exceed the amount of data that can be treated manually (Dupas et al., 2015; Kirchner & Neal, 2013). Nevertheless, without complete data series, it is not possible to accurately calculate total annual loads and the value of high-frequency monitoring is reduced to the observation of specific events that might not be representative of the overall system’s response.

Machine learning algorithms build models based on sample (training) data in order to make numerical predictions (regression models) or categorical predictions (classification models). Machine learning algorithms, such as trees, rules, support vector machines, and artificial neural networks, offer an advantage to linear methods when treating nonlinear problems such as concentration-discharge relationships. Although machine learning algorithms are powerful tools to post-process high-frequency water quality data, their use is still below their potential in many fields of environmental sciences (Liu et al., 2022). The relative low acceptance of machine learning in some environmental sciences may lie in reluctance to shift from a process-based approach to a data-based approach and the tradeoff between interpretability, performance, and complexity (Liu et al., 2022; Visser et al., 2022). Gap-filling of continuous water quality datasets is a so far unexplored, yet potentially powerful application of machine learning. Machine learning algorithms have been successfully applied for gap-filling in medical datasets (Shah et al., 2014), eddy-covariance evapotranspiration and CO_2_ flux data sets (Kang et al., 2019), soil moisture (Mao et al., 2019) and more recently also for daily streamflow time series (Arriagada et al., 2021). Most water quality applications of machine learning focus on predicting nutrient concentrations from catchment characteristics (e.g., Castrillo & García, 2020; Chen et al., 2020; Olson & Hawkins, 2012) or from other chemical parameters measured in conventional monitoring networks (e.g. Ha et al., 2020; Visser et al., 2022). Nevertheless, most of these studies focus on the forecasting performance and do not explore the limitations of using predictive data-based models (Tyralis & Papacharalampous, 2019).

The objectives of this study were as follows: (i) to evaluate six different machine learning models for gap-filling in a high-frequency NO_3_ and TP concentration time series, (ii) to showcase the potential added value and limitations of machine learning to interpret underlying nutrient transport processes, and (iii) to study the limits of machine learning algorithms for making predictions outside the training period. As case study, we used 4 years of high-frequency data from a ditch draining one dairy farm in the east of The Netherlands where the nutrient transport from the soil to the surface water were previously investigated (Barcala et al., 2020). We applied open source and popular data-science software such as WEKA (Frank et al., 2017) and R (R Core Team, 2020) for the data post-processing and model implementation. This study offers a valuable and novel example of how to use and interpret machine learning models for post-processing high-frequency water quality data.

## Materials and methods

### Field site and time series description

**Fig. 1:**
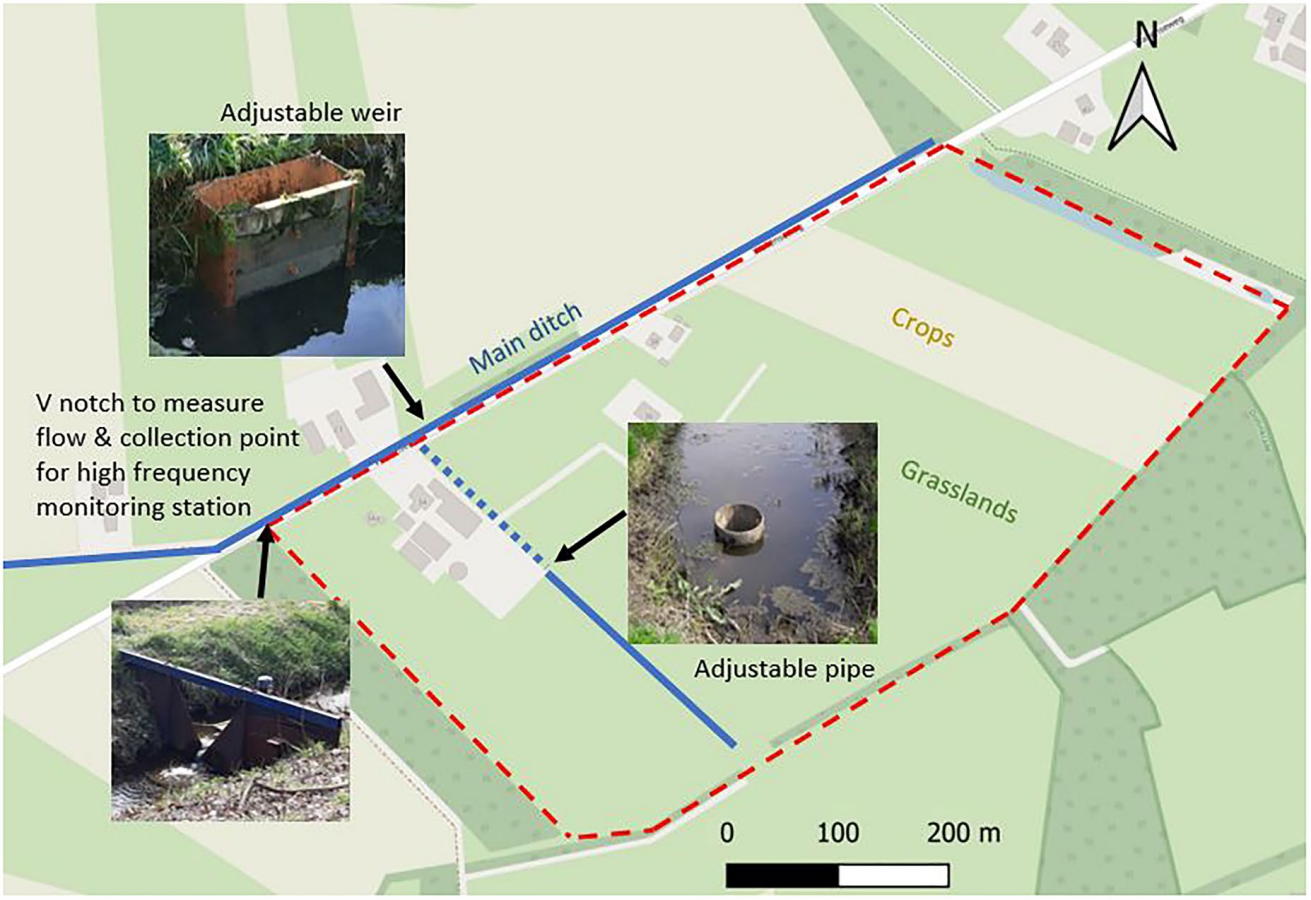
Farm layout (dashed red line) with water conservation measures implemented for the 2020–2121 season

The data was collected from a dairy farm near Winterswijk, the Netherlands (52.00131 N, 6.76112 E). The nutrient routes from the soil to the surface water were previously studied by Barcala et al. (2020). Manure is applied in the fields for fertilization between March and August. After measuring the crop productivity, the annual soil nutrient surpluses are calculated by the farmer. The topsoil is high in organic matter and has a 0.26 phosphorus saturation degree, meaning it can hardly retain more P. The water extractable P content (Pw) was on average 11.2 mg/kg in the topsoil, 1.5 mg/l just below the tillage zone (40–50 cm depth), and 0.1 mg/kg at 70–80 cm depth (Barcala et al., 2020). The average P in the topsoil is 2.630 kg/ha and the N in the topsoil is 565 kg/ha (Barcala et al., 2020). Below the topsoil, there is a Fe- and Al-rich sand layer. The farm is artificially drained by a main ditch that collects the water of the whole farm and runs parallel to the road in front of the farm. A secondary ditch runs perpendicular to the main ditch into the fields behind the farmyard. The northeast part of the fields has subsurface drainpipes draining into the most upstream part of the main ditch. The terrain is flat and surface runoff only occasionally contributed to the ditch discharge. Therefore, nutrients are transported mainly from the soil to the main ditch via lateral groundwater flow and the tile drains. During the summer months, the groundwater level falls below the ditch level and the main ditch falls dry. During this study, farmers were particularly affected by the extreme drought of 2018. Water shortage is a stress factor for crop growth and climate change is causing greater rainfall variability (Greve et al., 2021; Masson-Delmotte et al., 2021). To adapt against droughts, the farmer implemented different water conservation measures to control the groundwater level in the field before the start of the last drainage season. An adjustable weir was placed in the main ditch in front of the farm, and an adjustable pipe was installed in the side ditch behind the farmyard (Fig. 1).

At the end of the main ditch, we installed a v-notch weir and a high-frequency monitoring station was operative from 17 February 2018 to 7 June 2021. Every 15 min, TP (Phosphax Sigma autoanalyzer, Hach), turbidity (Solitax Sensor, Hach), and NO_3_ (Nitratax Sensor, Hach) were measured. About 80% of the total-N was in the form of NO_3_ in exploratory laboratory analysis. Furthermore, every 15 min, the discharge from the v-notch weir was calculated using a pressure gauge upstream from the weir and groundwater levels at the farm were monitored with a pressure gauge installed in a groundwater piezometer. As meteorological data may contribute to the prediction of the missing values, hourly rainfall and daily evapotranspiration data were downloaded from a meteorological station 12 km from the farm that belongs to the Dutch Royal Meteorological Institute Network (https://www.knmi.nl/nederland-nu/klimatologie, station 283). The hourly rain, and daily grass reference evapotranspiration, were linearly interpolated to have one value every 15 min using the *approxfun* function in R. All the times were taken to Dutch wintertime (GMT + 1). Time lags may vary depending on pre-event conditions such as groundwater levels, soil moisture, the location of the source (soil vs ditch sediment) and the magnitude of the event. Time lags were not included in the calculations but were estimated to be under 3 h. All the time series were quality checked, discharge measurements were checked based on manual measurements, and concentrations measurements were controlled based on laboratory measurements taken on routine visits every approximately 4 weeks. More detailed information about the field site characteristics and the high-frequency monitoring station can be found in Barcala et al. (2020).

Using the 2017–2018 values as a reference, the groundwater levels were on average 25 cm higher in 2020–2021 when the farmer implemented water retention measures (Table S1). In the year 2018 (2017–2018 season), TP correlated with turbidity (0.70) and NO_3_ correlated strongly with discharge and groundwater level (0.91, 0.86) as was already discussed in Barcala et al. (2020). However, these correlations became weaker in the following years, especially between TP and turbidity (Fig. S2). NO_3_ concentrations showed a similar temporal pattern to the groundwater levels but were shortly diluted during rain events (Figs. 3 and S3). Before starting with the selection of the machine learning models, we did some basic exploration of the available data available in the supplementary material. The N and P application to the fields are limited by the national Action Plans for the EU Nitrate Directive (Schroder et al., 2007). The manure applied targets at a 0 kg/ha P surplus. However, the crop growth can be limited by the water availability; in years with low rainfall less P was taken up, which resulted in a positive surplus. The average yearly N surplus was 142 kg/ha N, which falls just below the national average (160 kg/ha). Table 1 gives a quantitative summary of the drainage seasons.

**Table 1:** Summary of the nutrient surplus, first and last day of drainage, seasonal rainfall, evapotranspiration, and discharge

		All seasons	2017–2018	2018–2019	2019–2020	2020–2021
Nutrient surplus						
N surplus	(kg/ha)	568	126	209	167	66
P surplus	(kg/ha)	0	2	4	16	− 22
Drainage season characteristics						
First day drainage season	dd-mm-yy	17/02/18	17/02/18	23/12/18	18/10/19	1/12/20
Last day drainage season	dd-mm-yy	7/06/21	8/05/18	16/04/19	20/04/20	7/06/21
Rain	Total (mm)	1121	105	221	379	416
Evapotranspiration	Total (mm)	380	86	58	94	142
Rain–evap	Total (mm)	741	19	163	285	275
Discharge	Total (m^3^)	178,768	20,361	31,773	64,727	61,908
Data gaps						
Turbidity sensor	% Missing	5.9%	24.6%	1.8%	4.8%	1.6%
NO_3_ sensor	% Missing	5.6%	25.4%	1.5%	3.4%	1.9%
TP autoanalyzer	% Missing	33%	57%	34%	22%	34%
Number of instances	*N*	54,912	7776	11,040	17,952	18,144
Averages*						
Turbidity	(NTU)	9.40	2.06	14.1	4.89	13.4
Groundwater levels	(m)	− 1.28	− 0.989	− 0.916	− 0.858	− 0.769
NO_3_	(mg/L)	9.52	4.91	9.97	12.7	7.77
TP	(mg/L)	0.050	0.010	0.035	0.045	0.077

^*^To use as a reference for the average concentration, the Water Framework Directive target for surface waters is 2.3 mg/L for TN and 0.11 mg/L for TP (average summer concentrations)

### Data analysis

To calculate and compare accurate annual nutrient loads leaving the catchment, we needed to fill the missing NO_3_ and TP values of the high-frequency dataset. Table 1 shows the duration of each drainage season and the percentage of NO_3_ and TP missing data. To fill in missing data, we evaluated six machine learning algorithms and compared them to filling in the gaps with the mean. The measured data was split and 60% was used for training (calibration) and 40% for testing (validation). The data was randomly split three times to improve the statistical representation and robustness of the results by using three different seeds. Seeds ensure that the results are reproducible, dividing the data in the same way each time. We opted for a wide pre-selection of machine learning algorithms because one cannot know beforehand which will perform best for a specific problem. The performance depends on the available dataset and the defined problem, in our case the accuracy in filling missing data in high-frequency nutrient concentration time series. The Waikato Environment for Knowledge Analysis (WEKA) was used to preprocess the data and for evaluation of all Machine Learning models. WEKA is open software widely used for data mining programmed in Java (Frank et al., 2017). If it is not stated otherwise, the default parameter settings in WEKA were used. R studio (R Core Team, 2020) was used for data visualization and pre- and post-processing of the data. Six algorithms were pre-selected following the criteria that they were well documented and accepted, able to predict a numeric class (regression), and capable of handling missing data.

The pre-selected algorithms use different principles in order to build the models. Zero Rules (ZR) predicts the mean of the numeric class, it is used as a benchmark to determine if other algorithms perform better than filling the missing values with the mean. Multivariate linear regression (MLR) finds the best fit for a line between multiple independent variables and the output is an explicit equation. Sequential minimal optimization regression (SMO) is similar to a support vector machine but can solve regression problems. SMO solves analytically the smallest possible optimization problem at every step using two Lagrange multipliers that obey a linear equality constraint (Platt, 2008). K-nearest neighbor (kNN), also called instance-based learner, generates a prediction by first finding k instances in the training dataset which are closest to the value that we want to predict (Aha et al., 1991). K was set to 1 and we used the Euclidean distance. M5 Rules (M5R) combines rules with trees, it generates list of rules for regression problems using the “separate-and-conquer” strategy, in each iteration a tree is built, and the “best” leaf is made into a rule (Leman, 1997). Random forest (RF) grows an ensemble of trees and takes the average of the trees for the regression problem (Breiman, 2001), 100 trees were grown to maximal depth. Artificial neural networks (ANNs) are networks of linear classifiers (perceptrons), they implement a weighted decision given two hidden layers, we used 7 nodes or “neurons” in the hidden layer as this was equal to the number of nodes in the input layer (Wolpert, 1992).

To build the TP and NO_3_ models, we used time series of precipitation, evapotranspiration, groundwater levels, discharge, turbidity, and NO_3_ or TP, respectively. Seasonal changes as the manure surplus and the implementation of water retention measures are not included as predictors. To evaluate and select the best model, we used the coefficient of determination (*R*^2^), the mean absolute error (MAE), and the root mean square error (RMSE) between the measured and the predicted values of the test subset. The models were done for each drainage season (2017–2018, 2018–2019, 2019–2020, 2020–2021) and for all the seasons together (2017–2021). The seasons are defined as the time during the year when there is water discharge in the ditch. Separate models were preferred to one single model to evaluate the model response to different yearly features that are not considered as predictors, such as year-to-year variations in total rainfall, nutrient surpluses, and the implementation of water conservation measures in the 2020–2021 drainage season.

To study the performance of predicting nutrient concentrations we evaluated two scenarios. First, the 2018–2019 model was used to predict the 2019–2020 measurements, and second, the 2019–2020 model was used to predict the 2020–2021 measurements. Both predictions used the input variables (rain, evapotranspiration, groundwater, discharge, turbidity, and NO_3_ or TP) of the season we wanted to predict. The first scenario represents the prediction with no system changes and the second represents the prediction with changes (water retention measures). This way we evaluate if the model can predict system changes outside the training window. Both predictions were also done with the all seasons’ (2017–2021) model to assess if the model could represent system changes inside the training window. Although the retention measures are not incorporated into the model, the groundwater levels measured were and they were higher on the last season.

The feature importance (also called permutation variable importance metrics) allows to weight the influence of each input variable in the prediction, improving the interpretability of the results. The feature importance is calculated as the percentual increase in predictive error of not including one variable as compared to the out-of-bag rate with all other variables intact (Breiman, 2001). The feature importance was used to interpret which variables are most relevant for the prediction outcomes. An extra random variable was included in the feature importance calculations as a benchmark. If any variable was equally or less important than the random variable, then it would not contribute for the prediction. The importance values are related to the output magnitude of the predicted variable; therefore, the feature importance was normalized to 1 to facilitate the comparison. Without this normalization step, the NO_3_ predictors would have a higher feature importance values than those for TP. The 2017–2018 season is only used for gap filling and not for future predictions or for variable feature importance calculations because it is not a full drainage season (it starts in half February). Lastly, after filling the missing data with the best performing model, the total loads of NO_3_ and TP were calculated for each season by multiplying the concentrations by the discharge. Total loads were also calculated for the predictive models.

## Results

### Filling in missing data

**Fig. 2:**
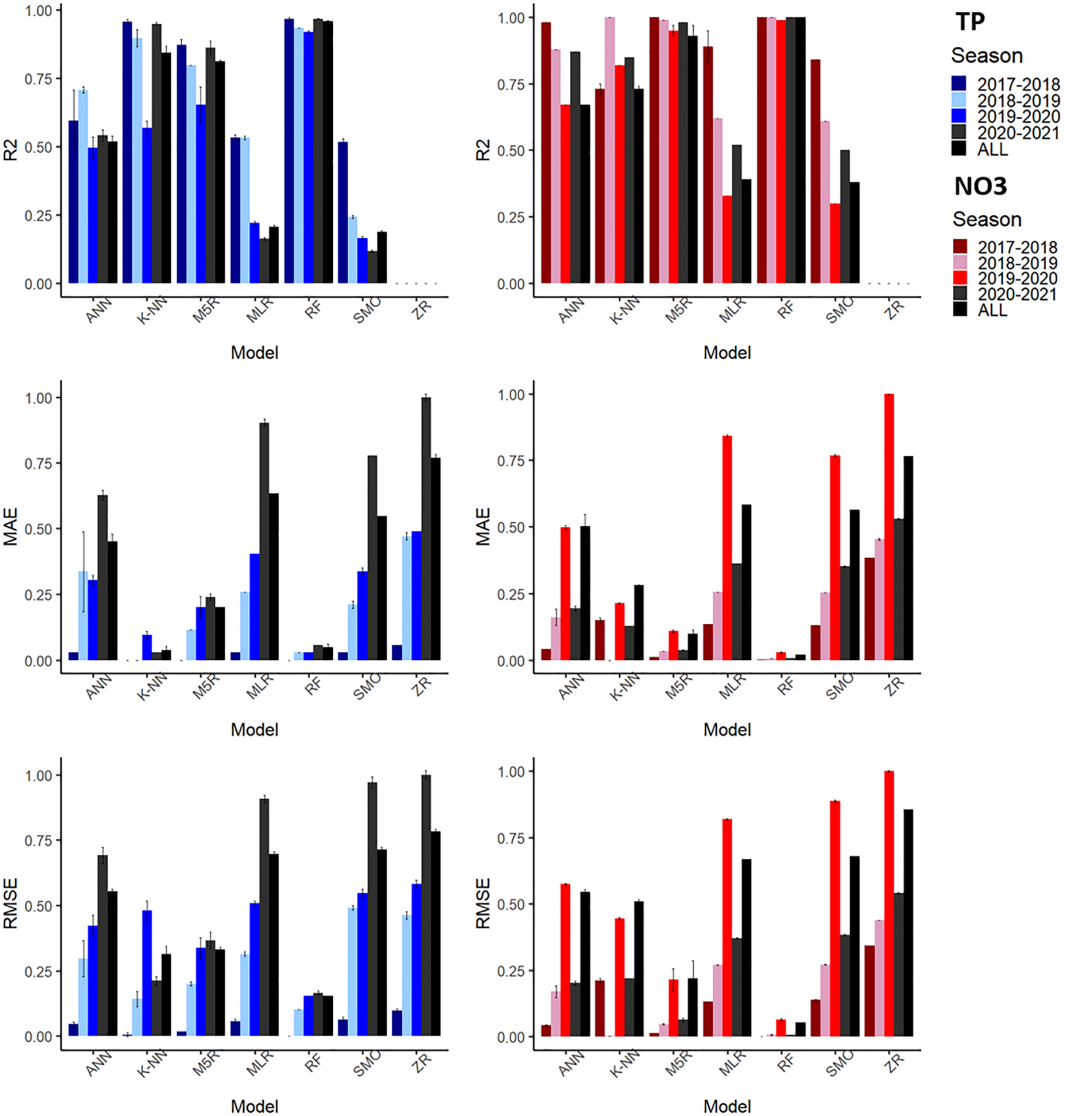
Performance of the different machine learning models in the test set. Average of *R*^2^, MAE, and RMSE for the 3 seeds used. The standard deviation is shown with the error bars. Models: artificial neural networks (ANNs), K-nearest neighbor (K-NN), M5 Rules (M5R), random forest (RF), sequential minimal optimization (SMO), Zero Rules (ZR). MAE and RMSE were normalized to facilitate comparison

**Fig. 3:**
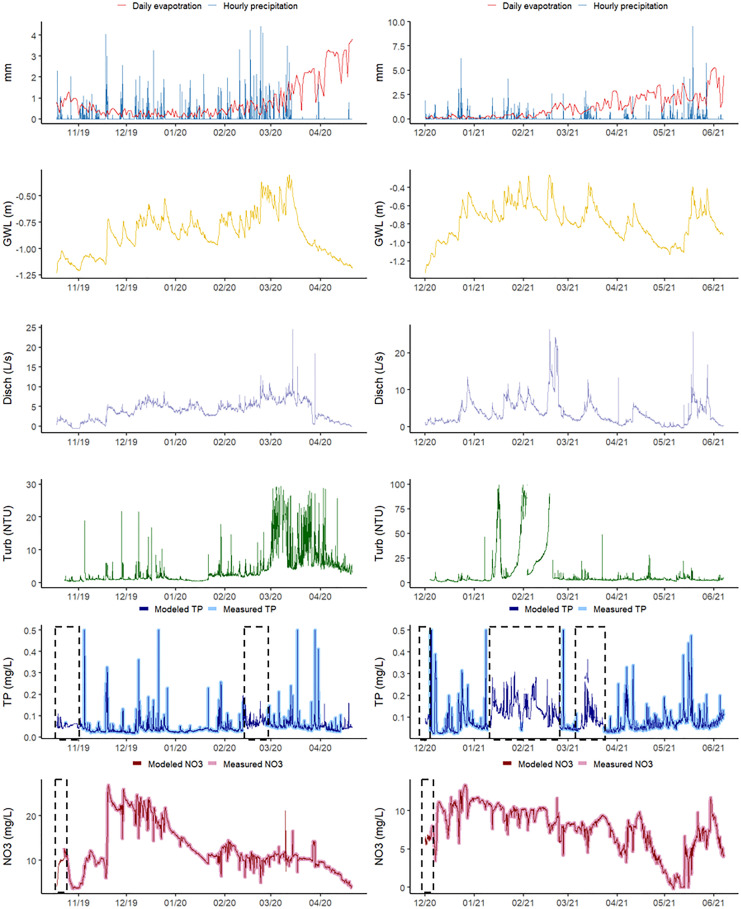
Measured and modeled time series for the 2019–2020 and 2020–2021 season. The nutrient measured data was plotted thicker to see it behind the model (random forest). Gaps in the data are indicated with a dashed box. The groundwater levels are relative to the ground level

The TP autoanalyzer had a larger amount of missing data, grouped in 2 to 4 large gaps per season. Besides 2018, the amount values missing from the NO_3_ sensor were very low and concentrated at the beginning of the season. The summary of the *R*^2^, RMSE, and MAE for three test subsets for the different NO_3_ and TP season models are shown in Fig. 2 (values and computation times are shown in Tables S2 and S3). For each drainage season, the random forest model had the best fit (*R*^2^ ~ 0.99 for NO_3_ and 0.96 for TP) with low computation times and was therefore selected to fill in the missing values. Figure 3 shows the complete measured and modeled time series together with the input variables for the 2019–2020 and 2020–2021 seasons (seasons 2017–2018 and 2018–2019 are in Fig. S3). Random forest gave consistently very good results for each seasons’ model, while other algorithms showed larger differences in performance from season to season. Following random forest, k-nearest neighbor, and M5 Rules had a good performance for all seasons’ (*R*^2^ ~ 0.84 and 0.81 for TP and 0.73 and 0.92 for NO_3_, respectively), yet they had poorer results for TP in the 2019–2020 season (*R*^2^ ~ 0.59 and 0.65, respectively). M5 Rules gave, for little extra computation time, a very good performance in the all seasons’ model and has the advantage that the output of the model are explicit rules that could be interpreted. However, as the problem was complex, more than 190 rules were obtained, which makes interpretation very difficult. Artificial neural networks came in fourth place with a lower performance in the all seasons’ model (*R*^2^ ~ 0.67 for NO_3_ and 0.50 for TP). Sequential minimal optimization obtained even lower results than artificial neural networks (*R*^2^ ~ 0.38 NO_3_ and 0.19 TP) and the longer times needed to build and validate the model are a mayor disadvantage. For example, for the all seasons’ model, the sequential minimal optimization model took 46 h computing time to train and test the NO_3_ data series while random forest took only 2 min. Multivariable linear regression offers the benefit of having an explicit equation as output, but the trade-off is a lower performance (*R*^2^ ~ 0.39 for NO_3_ and 0.21 for TP; MAE > mean). Only in 2018 when NO_3_ was strongly correlated to discharge and groundwater levels the results for the multivariable linear regression were very good (*R*^2^ ~ 0.89). Nevertheless, the correlation was almost the same as doing a simple one variable linear regression with the discharge and this relationship was not maintained through the years (Fig. S2).

### Future predictions

**Fig. 4:**
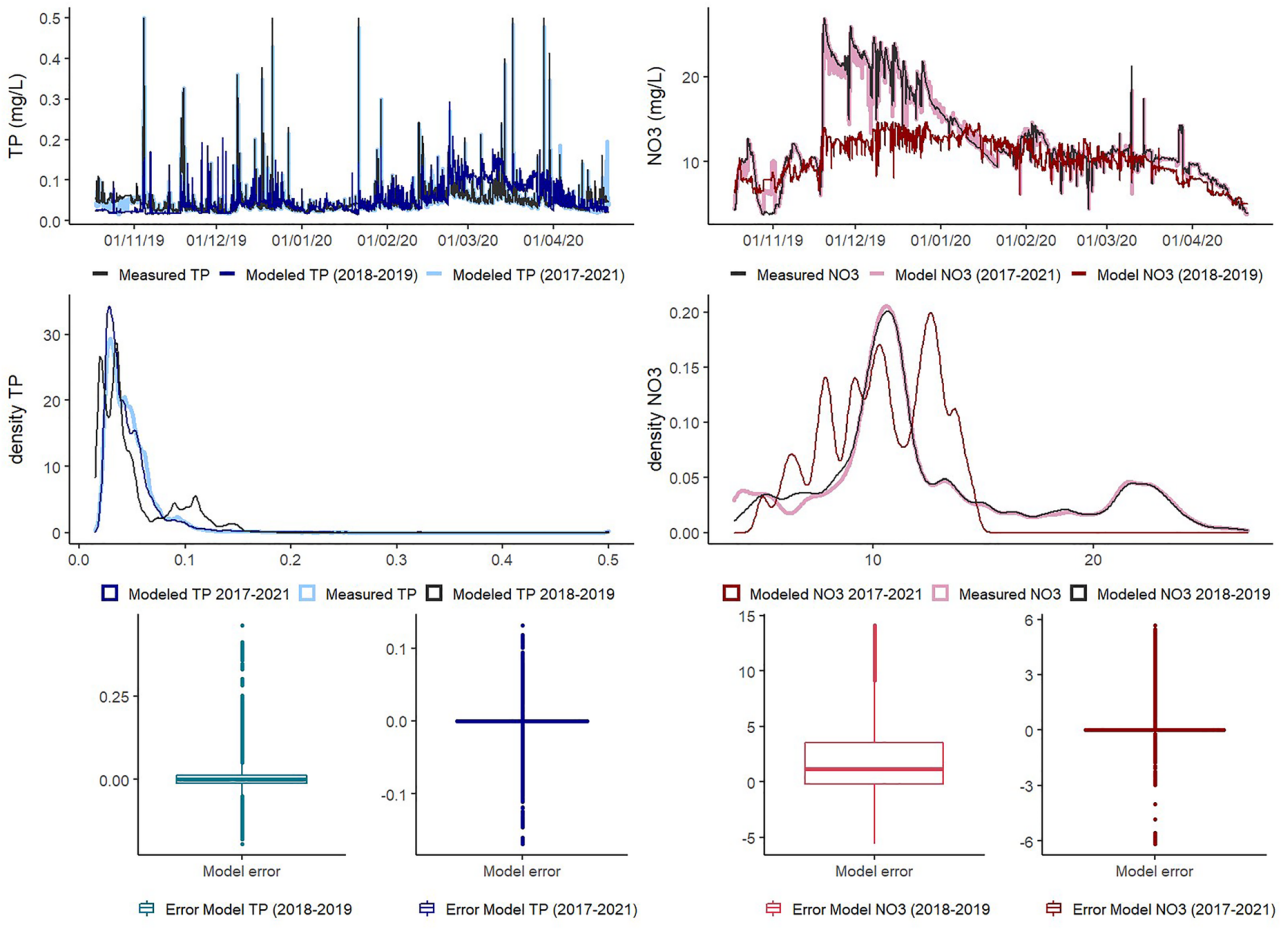
Prediction of 2019–2020 measured concentrations using 2018–2019 and 2017–2021 models. Concentrations’ time series (top), density distribution of values (middle), and box plots of the error (measured–modeled) (bottom)

First, we compared the predictive performance between the 2019–2020 measured data and the prediction of the same season using the 2018–2019 and the all seasons’ random forest models (Fig. 4). This prediction illustrates the random forest performance outside the training period without system changes (besides manure surplus). The *R*^2^ using the TP predictive 2018–2019 model was only 0.41 (MAE 0.02 and RMSE 0.04), fewer and lower TP peaks were obtained but the baseline concentrations were reproduced, except for some baseline overestimations in March. For NO_3_, the largest difference in concentrations was from the end of November to January, when there is an increase in the measured NO_3_ values that were not predicted by the model, still the *R*^2^ was 0.75 (MAE 3.02 and RMSE 4.40), as the model performs well outside this window. The NO_3_ load using 2018–2019 model was 723 kg while the measured load was 885 kg. TP exported using 2018–2019 model was 4.1 kg while the measured load was 3.3 kg.

**Fig. 5:**
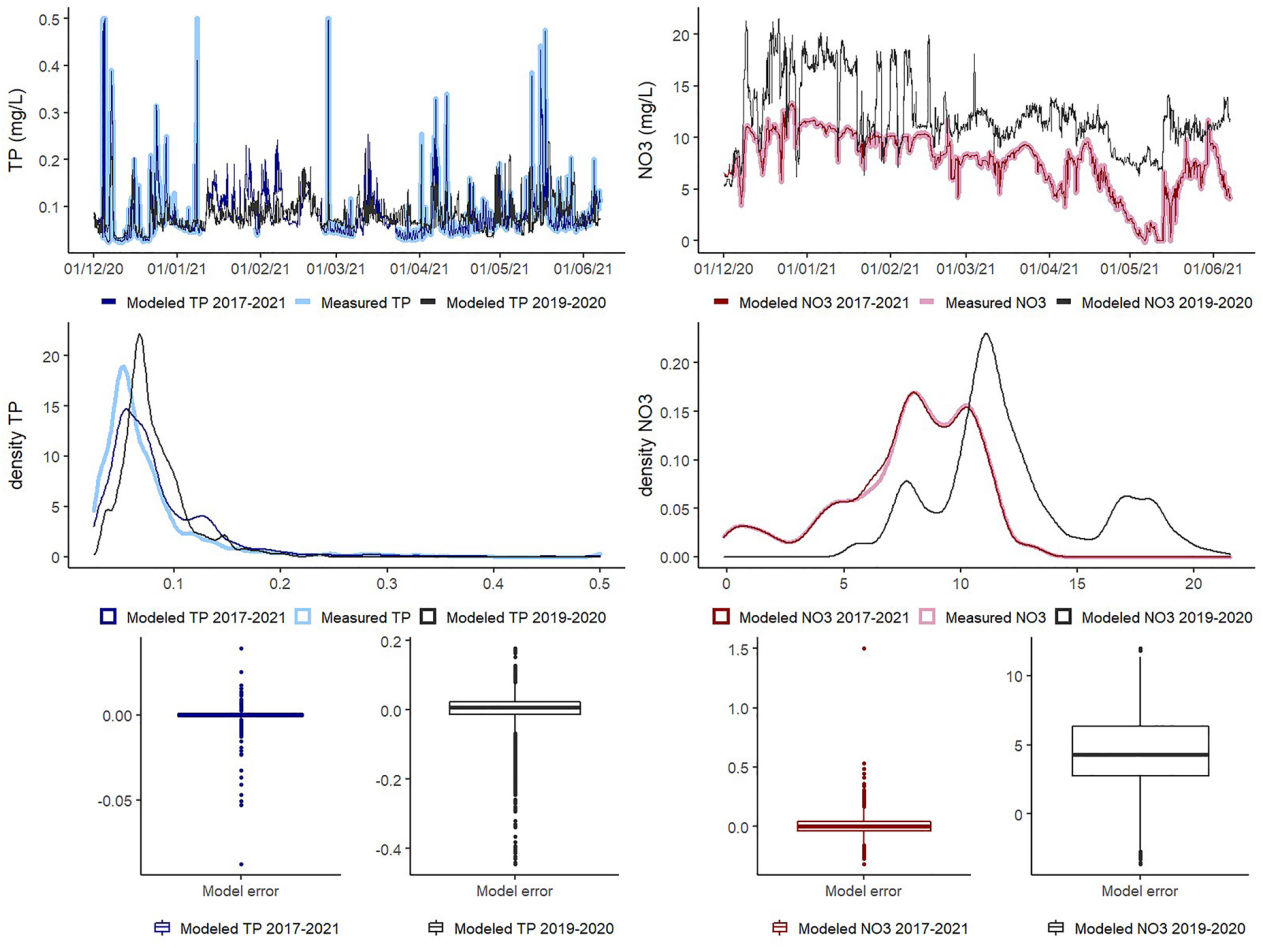
Prediction of 2020–2021 concentrations using 2019–2020 and 2017–2021 model. Concentrations’ time series (top), density distribution of values (middle), and box plots of the error (measured–modeled) (bottom)

Second, we compared the predictive performance between the 2020–2021 measurements and the prediction of the same season using the 2019–2020 and the all seasons’ random forest models (Fig. 5). The predictions represent how suitable is random forest to capture system changes (water conservation). The performance of the 2019–2020 model TP predictions was poor (0.09 *R*^2^, 0.034 MAE, and 0.057 RMSE) while the all seasons’ model performed well (0.96 *R*^2^, 0.001 MAE, and 0.003 RMSE). In the case of NO_3_, the *R*^2^ of the 2019–2020 model with the measured values was 0.44 (4.53 MAE and 5.15 RMSE) while with the all seasons’ model it was 0.99 (0.050 MAE and 0.070 RMSE). The distribution of the error of the all seasons’ model was always around zero and the differences with the measurements were mainly in the outliers. Despite the very good results for gap-filling within the training period, the predictive performance of random forest outside the training period was poor. The total TP load using the 2019–2020 model predictions was 5.57 kg while the measured load was 6.55 kg. On the other hand, the 2019–2020 model overestimated the NO_3_ load at 760 kg, while the measured load was 534 kg.

**Fig. 6:**
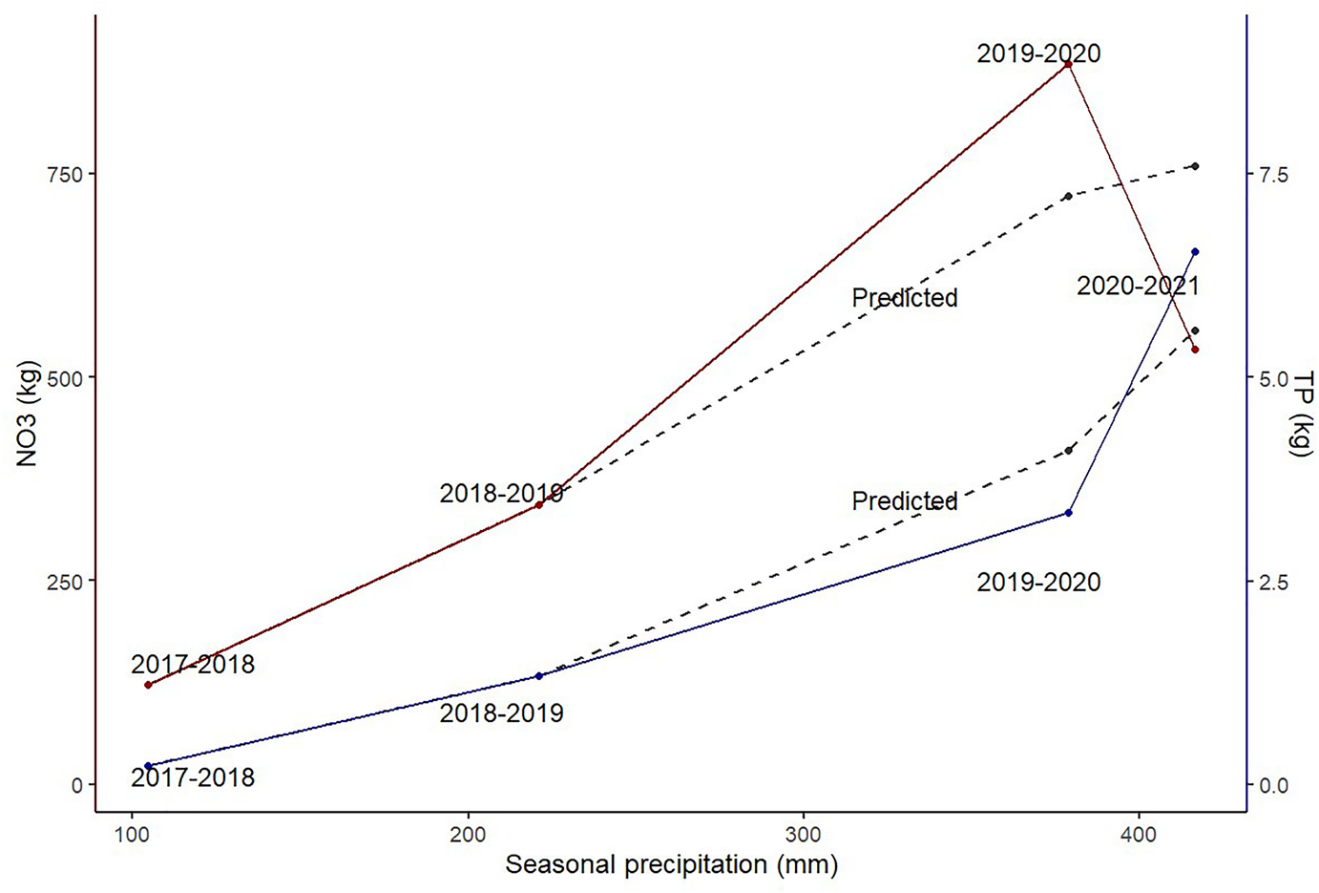
Total loads of NO_3_ and TP per season against the seasonal precipitation during drainage period. The predicted loads for the last seasons with the 2018–2019 and 2019–2020 model are included in gray

**Fig. 7:**
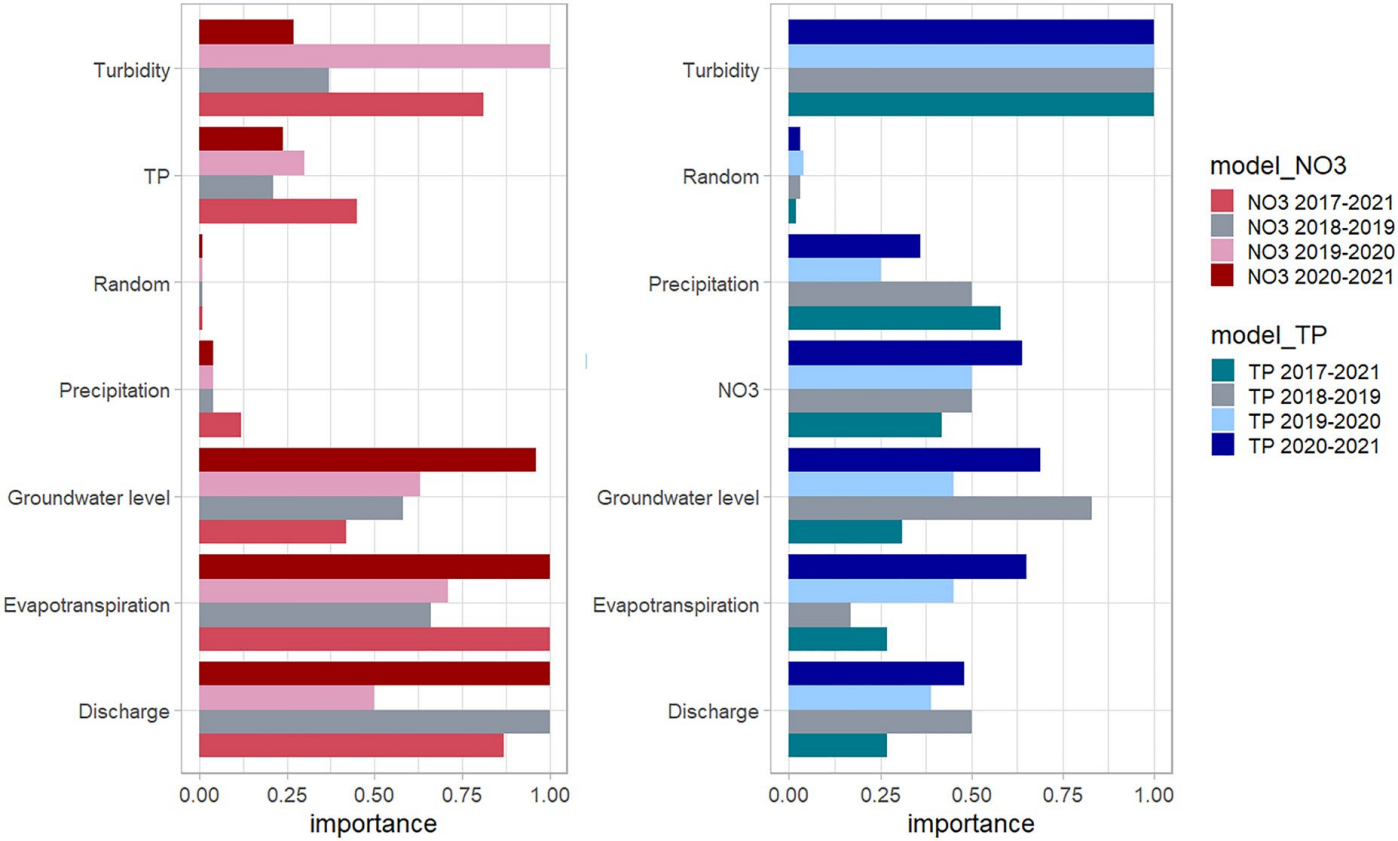
Relative feature importance of the variables in the random forest models of the 2017–2021, 2018–2019, 2019–2020, and 2020–2021 seasons

During the first three seasons the total nutrient loads appears to have increased with the rainfall. A change in this trend is observed for the last season after the water conservation measures were implemented. Although the predicted loads differ from the measured loads for 2020–2021, the change in trend is to some extent captured by the model (Fig. 6). The dryer years with lower leaching may have resulted in accumulation of nutrients in the topsoil that were later released in the wetter years. All measured variables had between four and a hundred times the importance of the random variable introduced (Fig. 7). Turbidity had the highest feature importance in most models (except the 2018–219 and 2020–2021 NO_3_ models).

## Discussion

### Using machine learning models to fill in missing data

The first objective of this study was to evaluate six different machine learning models for gap-filling in a high-frequency NO_3_ and TP concentration time series. Random forest had the best performance for both NO_3_ and TP with a constantly high *R*^2^ (> 0.92) and low MAE and RMSE for every randomly selected testing set and application period. The short computing times are another advantage for using random forest for gap filling. The random forest gap-filling model could reproduce short-term trends in the time series as the TP peaks after rain events, NO_3_ dilution after rain events, and NO_3_increase with increase in the groundwater levels. The good results of the 2017–2021 model show that the random forest algorithm can also largely incorporate system changes within the training period, such as the introduction of water conservation measures and different soil nutrient surpluses. We observed that the NO_3_ models performed systematically slightly better than the TP models. This may be caused by the larger proportion of missing values in our TP time series. Kang et al. (2019) and Zhang and Thorburn (2022) also reported a reduction in model performance when the amount of missing data is larger as this reduces the size of the training set. Another reason could be the relatively smooth behavior of NO_3_ concentration dynamics compared to the spikier TP patterns.

Other comparative studies have also found random forest to perform better than linear regression and other machine learning algorithms in problems related to water quality (Castrillo & García, 2020; Ha et al., 2020; Shen et al., 2020; Visser et al., 2022). Methods such as artificial neural networks that here had a low performance have shown very good results in other studies (Astuti et al., 2020; Chen et al., 2020; Daliakopoulos & Ioannis, 2016; Dastorani et al., 2010; Kim et al., 2020; Najah et al., 2009, 2013). Nevertheless, in other comparative studies, they have also underperformed random forest (Bedi et al., 2020; Chen et al., 2020; Kim et al., 2020; Qiao et al., 2021; Visser et al., 2022). The low performance of multivariable linear regression can be explained by the nonlinear relationships between hydrological variables and concentrations. Multivariable linear regression has shown low performance in other nonlinear problems such as epidemiological studies (Shah et al., 2014). As there is no one-fits-all model, we recommend the used approach of evaluating different algorithms and seasonal performance to select the most robust model.

Conventional approaches for dealing with missing data included not considering the missing data or substituting the missing data with the mean (Tyralis & Papacharalampous, 2017). Not including the missing data makes calculations of total annual loads uncertain, especially because short, extreme rain events can account for a large portion of the yearly nutrient load export (Rozemeijer & Van der Velde, 2014). Substituting the missing data with the mean was done as a benchmark (Zero Rules model) and it underperformed all other methods having the highest MAE and RMSE. It has been already shown that statistical gap filling methods usually underperform machine learning methods (Zhang & Thorburn, 2022). Another gap-filling method includes stepwise linear regression; this approach has exhibited a good treatment of the missing data (Rozemeijer et al. (2010a, b), *R*^2^ 0.74), but it requires a more a complex and time-consuming analysis of the time series than machine learning models as random forest. In our previous publication (Barcala et al., 2020), TP was correlated with NO_3_ for gap-filling in the 2018–2019 season; this lead to a lower load estimations, 0.96 kg for TP and 282 kg for NO_3_ compared with loads obtained with the new method, 1.33 kg for TP and 344 kg for NO_3_. Random forest, and other machine learning algorithms, are underused tools in water quality studies. We encourage their application for gap-filling because of the good performance, short calculation times, and the availability of open source packages in R and user-friendly software like WEKA. Beside gap-filling, algorithms like random forest could also be useful for similar applications as real-time anomaly detection in sensors and autoanalyzers which could support system maintenance, for example by comparing the incoming data with one-step ahead predictions to detect anomalies and trigger a warning message.

### Process interpretation

Our second objective was to show potential added value of machine learning to interpret underlying nutrient transport processes. In most cases, the high number of trees in random forest models makes their physical interpretation difficult or impossible (Tyralis & Papacharalampous, 2019). The feature importance represents the information gain of including each variable in the model and could be indicative for the influence of variables on physical processes. As a first observation, all predictor variables in our data set contributed to the information gain for all drainage seasons. This was indicated by the higher relative feature importance values compared to the random variable introduced (Breiman, 2001; Doshi-Velez & Kim, 2017). Therefore, it is not advised in this case to remove variables as all of them contributed to the prediction. Besides the feature importance, other approaches for process interpretation include to evaluate the variable coefficients obtained by multivariable linear regression for process interpretation together with the results of “less transparent” models as random forest (Visser et al., 2022). However, we would not recommend this approach for case studies as this one where results obtained with multivariate linear regression are poor. Instead, a similar sensitivity analysis to the variable feature importance can be done for other machine learning algorithms by training the models without one input variable at a time and evaluating the impact on the model’s results.

For NO_3_, the feature importance values were quite different between the 2019–2020 and 2020–2021 seasons. In the 2019–2020 season, turbidity was the most important predictor, although groundwater levels, evapotranspiration and discharge also contributed significantly. In the 2020–2021 season, groundwater levels, evapotranspiration, and discharge were the most important variables, but turbidity did not rank high. The connection between groundwater levels, discharge, and NO_3_ losses was described before by Rozemeijer and Broers (2007) and for this field site by Barcala et al. (2020). With higher groundwater levels, a larger relative contribution of shallow NO_3_-rich groundwater flow routes (including tube drain discharge) towards surface water increases the NO_3_ concentrations while rain events dilute the concentrations. The NO_3_ concentrations reached up to 25 mg/L NO_3_-N after an increase in the groundwater level in mid-November 2019. During the second part of the drainage season, the mineral N residue in the soil was depleted and the NO_3_ concentrations decrease to around 10 mg/L. Turbidity was also high in the second part of the season and splitting the trees by turbidity resulted in a positive information gain. In this case, the predictive power of turbidity does not seem to have a direct process-based explanation.

For TP, turbidity has the highest relative feature importance values in all models. This relation is directly linked to the role of sediment transport in TP concentration dynamics. As described by Barcala et al. (2020) and Baken et al. (2015), iron and phosphorus from groundwater form iron(hydr)oxides which precipitate at the ditch bottom. During steady hydrological conditions, a P-rich sediment layer builds up. This sediment is transported during the next discharge event, causing a peak in both turbidity and TP. The data gaps in 2020–2021 coincided with the highest turbidity peaks, and although it is likely, it is not possible to asses if there was an underestimation of TP peaks during this period. Furthermore, almost no high TP peaks were predicted with the 2019–2020 model when compared with the 2020–2021 measured series. Shen et al. (2020) observed that high values were underestimated when using random forest to predict N and P concentrations in streams. The high skewness of the data was offered as explanation. Surprisingly, the TP export increased in the last season (2020–2021) despite the negative P surplus. The groundwater level increased in importance in the 2020–2021 model. The risk of mobilization of phosphate (and heavy metals) with the introduction of water conservation has been proposed theoretically before (Rozemeijer & Griffioen, 2004; Schoumans & Groenendijk, 2000), but to the best of our knowledge, it has not been directly measured yet. The topsoil had higher P and lower Al and Fe content, therefore watering the topsoil may have increased the risk of P leaching. In a recent data-based model of NO_3_ leaching from agricultural soils across the Netherlands comparable trends were found (Spijker et al., 2021), the TP concentrations were inversely correlated with NO_3_ emissions and TP was important for the prediction of NO_3_ concentrations. They hypothesized that the high TP concentrations were a proxy for high groundwater levels (which were not included in that model), and that areas with high groundwater levels had higher denitrification rates and therefore lower NO_3_ concentrations. In addition, Skidmore et al. (2022) has recently shown that extreme rain events increase the TP loads from agriculture.

Overall, machine learning can support process interpretation but justification of findings by other methods is needed, as the feature importance can be related to indirect links. Finally, turbidity was the variable that represented the largest information gain. Arriagada et al. (2021) and Fox et al. (2017) showed that random forest performance increased with the number of input variables used. Sensors are cheaper than autoanalyzers, require less maintenance and do not use reagents. The sensor data can then be trained to fill in data gaps in more complex equipment as TP autoanalyzers. This reason largely justify adding sensors (such as for turbidity, conductivity, dissolved oxygen, or temperature) next to autoanalyzers at high-frequency monitoring stations. Moreover, if a TP autoanalyzer is removed, the combination of continued cheap sensor measurements, low-frequency conventional TP sampling, and the previously trained RF model could still produce accurate continuous TP concentration time series for the site.

### Using machine models for forecasting

The third objective of this study was to show the limits of machine learning algorithms for making predictions outside the training period. As reported by Tyralis and Papacharalampous (2019), the most important limitation to data-based models is that they should not be generalized to predict new processes or changes in unaccounted variables that were not covered by the training data set. Moreover, Kang et al. (2019), observed that interannual variations in nutrient loads are caused by year-to-year system changes for example in manure application, crop rotation, and cultivated area percentage, which were not fully captured by their random forest models. One disadvantage of random forest is that a small change in the data set, caused for example by different manure surpluses, can lead to a large change in the structure of the optimal decision tree. Unfortunately, the soil nutrient surplus is calculated at the end of the season; therefore, it is not possible to use it as a predictor for result forecasting.

Although for each season the all seasons’ model and the model of the season had similar *R*^2^, MAE, and RMSE, the trees of each model were built splitting by different variables. This is why although the fitting was good for testing sets contained in the training period, it did not show such a good predictive performance outside that period. For example, the high feature importance of the variable turbidity to predict NO_3_ in the 2019–2020 season is likely because during the first half of the season NO_3_ was high and turbidity was low while in the second half the opposite happened, NO_3_ was low and turbidity was high. Whereas in the previous (2018–2019) season, this negative correlation did not occur the importance of turbidity in the prediction was low. When we see the feature importance of the variable turbidity for the whole period (2018–2021), it is larger than for the 2018–2019 and 2020–2021 seasons. Therefore, the longer the data set used to build the model, the more likely it is that more processes that directly or indirectly affect the prediction are taken into account.

Despite both predictive models underperform the gap-filling results, the 2018–2019 model does a better job reproducing the 2019–2020 data than the 2019–2020 for the 2020–2021 data. Especially the NO_3_ prediction with the 2019–2020 gives quite fair results. The relatively low N surplus in 2020–2021 may explain the differences in NO_3_ loads obtained with the predictive model. In the last season, water retention measures were introduced and the nutrient N and P surpluses were lower, both changes were not directly introduced in the model as predictors but indirectly through the groundwater levels and the water quality data. Therefore, the groundwater level measurements were not enough to explain the system changes outside the training window. Nevertheless, the all seasons’ model performed very well for each of the individual seasons, including the 2020–2021 season. Random forest can cover the system changes as long as they occur within the training period. The presented example shows that historical trends are no guarantee for future performance and that emerging processes may not be accurately predicted by the model. This highlights the need for cautious interpretations of machine learning model predictions and for keeping the models up to date using longer data sets to improve the robustness of the models.

It is important to notice that the issue regarding the uncertainty of predictions outside the training period is not likely to be caused by overfitting. Overfitting occurs when the performance of the model is good in the training set but not in the testing set. The training set was 60% of the data and it was randomly divided three times. The resulting random forest models were robust, with good results on all different validation sets and application periods. Moreover, we used a high-quality dataset with seven variables and about 18,000 instances in the last two seasons. For further studies, we recommend evaluating ways to include low-frequency annual system changes in data-based models and quantifying the impact of the amount of missing data for the model performance.

## Conclusions

- Random forest was the best out of six machine learning algorithms to fill in missing data, with an *R*^2^ higher than 0.92 for all test sets. Random forest could effectively reproduce nonlinear processes as concentration-discharge relationships and represent system changes that were considered in the training set.
- Machine learning may support process interpretation, but justification of findings by other methods is needed. Accounting for changes in the groundwater levels was not enough to accurately predict system changes as the water conservation caused changes in the nutrient processes. After water conservation, higher groundwater levels resulted in more TP leaving the farm despite the negative P surplus and was represented by an increase of the relative feature importance of the groundwater level variable. This effect was likely related to higher desorption from the topsoil layers. For NO_3_, the variable feature importance values were caused by indirect links and were not directly related to processes.
- The incorporation of subsidiary sensors can pay-off in monitoring stations with more sensitive autoanalyzers. Here, the turbidity showed the largest information gain in predictions.
- Random forest predictions outside their training period can be uncertain. Keeping the machine learning models up to date with newly retrieved data increases the reliability of the predictions.
- Similar to gap-filling, random forest can be used for anomaly detection in monitoring stations.

The supplemental material includes the full data series, basic statistical exportation of the data series, model results including time calculations, plots of the modeled and measured data for 2017–2018 and 2018–2019 seasons, plots of the cumulative load for each season, scatter plots of measured vs modeled data with Random Forest for every season.

## Supplementary Information

Below is the link to the electronic supplementary material.

Supplementary file1 (DOCX 464 KB)

## Data Availability

The raw data used in this study is available at https://github.com/victoriabarcala/Huppel.

## Abbreviations

ANN: Artificial neural network
kNN: K-nearest neighbor
M5R: M5-Rules
MAE: Mean average error
MLR: Multivariable linear regression
N: Nitrogen
NO_3_: Nitrate
P: Phosphorus
*R*^2^: Coefficient of determination
RF: Random forest
RMSE: Root mean square error
SMO: Sequential minimal optimization
TP: Total phosphorus
ZR: Zero Rules
