# Supplementary material for: Value and limitations of machine learning in high-frequency nutrient data for gap-filling, forecasting, and transport process interpretation
Source: Environ Monit Assess. 2023 Jun 27;195(7):892. doi: 10.1007/s10661-023-11519-9 (PMC10299926; doi:10.1007/s10661-023-11519-9)
Supplement: Supplementary file 1 — Supplementary file1 (DOCX 464 KB) [file 10661_2023_11519_MOESM1_ESM.docx]

Supplemental Material

Value and limitations of Machine Learning in high-frequency nutrient data for gap- filling, forecasting, and transport process interpretation

Victoria Barcala^1^, Joachim Rozemeijer^2^, Kevin Ouwerkerk^2^, Laurens Gerner^4^, Leonard Osté^1^

^1^ Deltares, Unit Inland Water Systems, Daltonlaan 600, 3584 BK Utrecht, The Netherlands

^2^ Deltares, Unit Subsurface and Groundwater Systems, Daltonlaan 600, 3584 BK, Utrecht, The Netherlands

^4^ Water Board Rijn and IJssel, Liemersweg 2, 7006 GG Doetinchem, The Netherlands

The supplemental material includes the full data series, basic statistical exportation of the data series, model results including time calculations, plots of the modeled and measured data for 2017-2018 and 2018-2019 seasons, plots of the cumulative load for each season, scatter plots of measured vs modeled data with Random Forest for every season. The data series are available on <https://github.com/victoriabarcala/Huppel> .

**Full high-frequency datasets per season with measured TP and NO_3_ with data gaps**


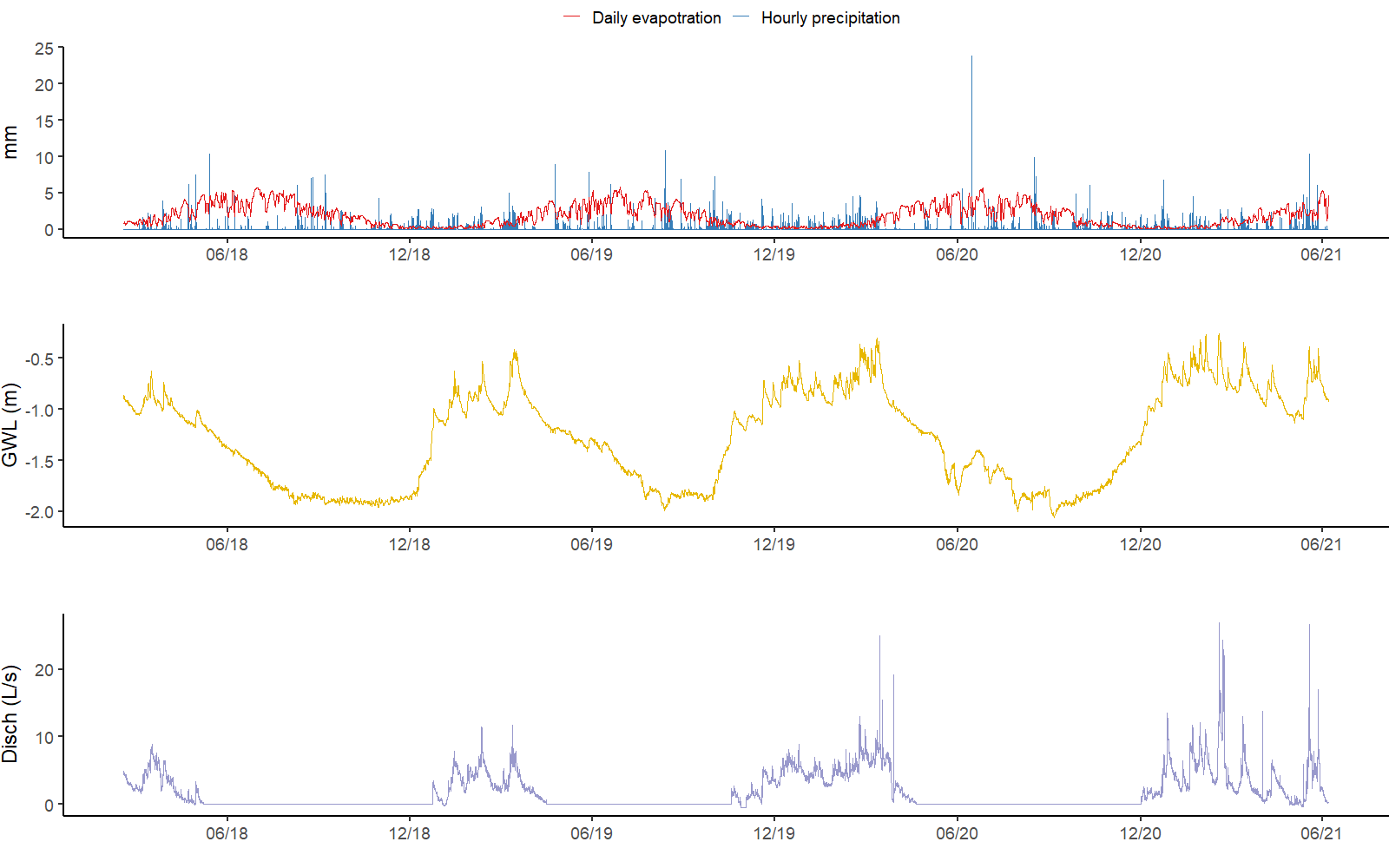


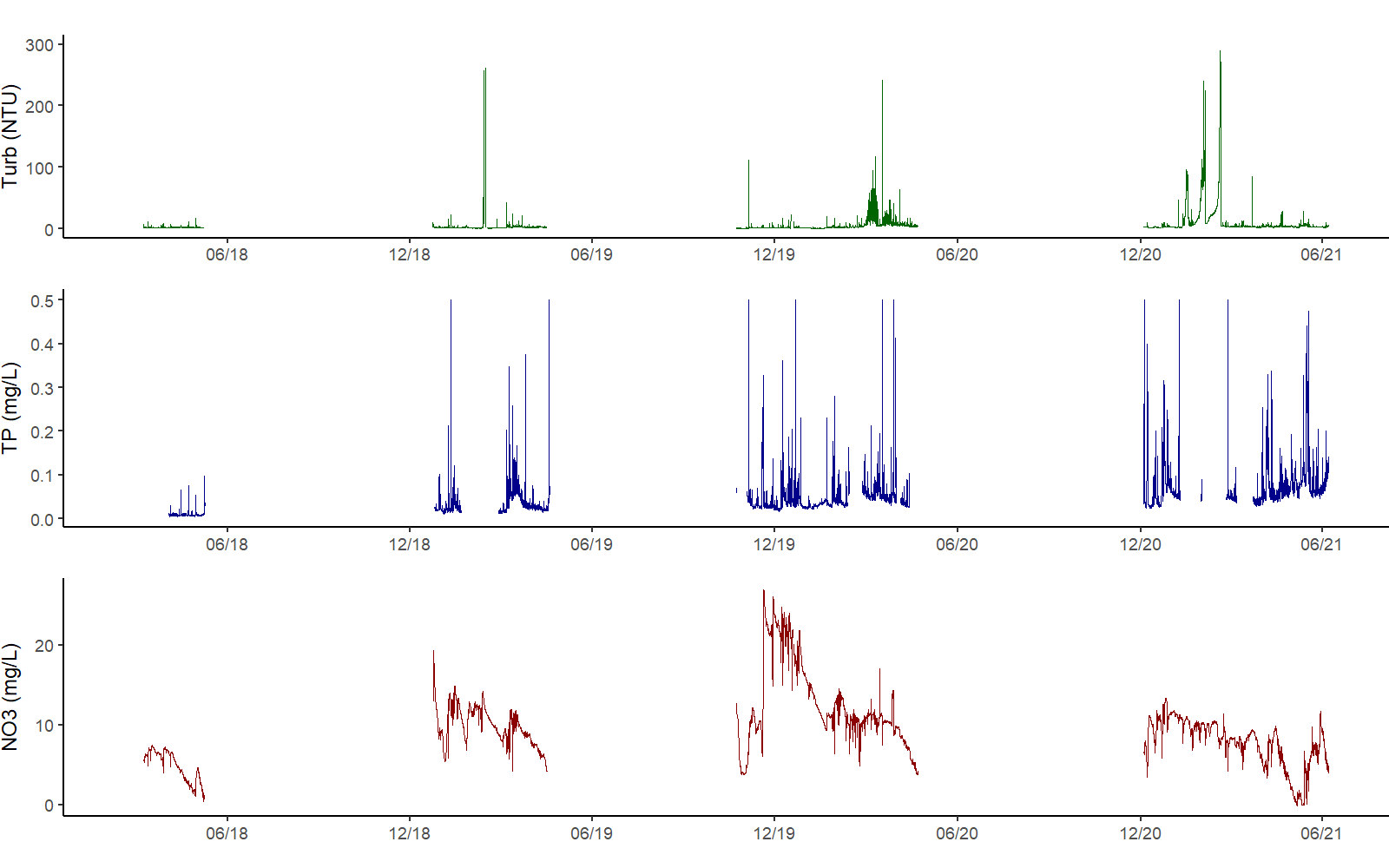


Figure S1 Measured time series with gaps in the data

| **2017-2018 season correlation matrix** | **2018-2019 season correlation matrix** |
| --- | --- |
| 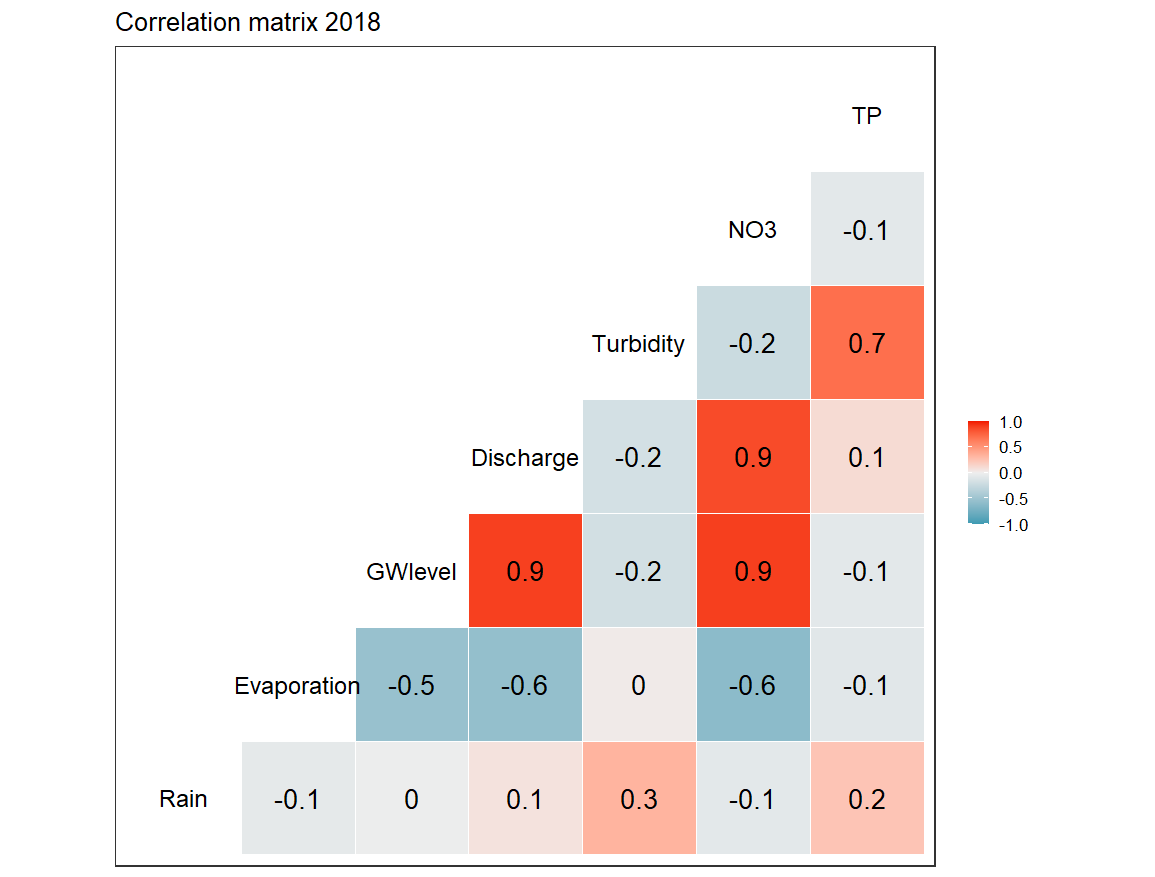 | 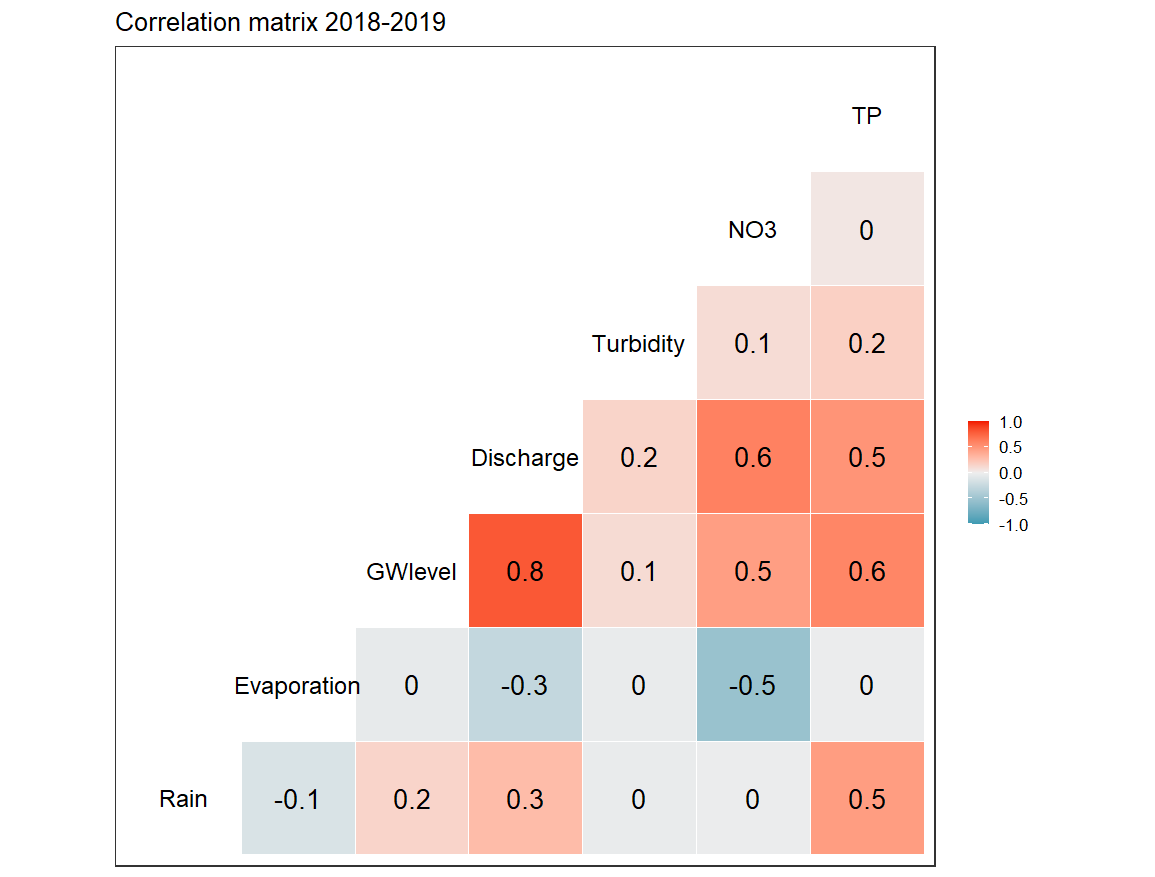 |
| **2019-2020 season correlation matrix** | **2020-2021 season correlation matrix** |
| 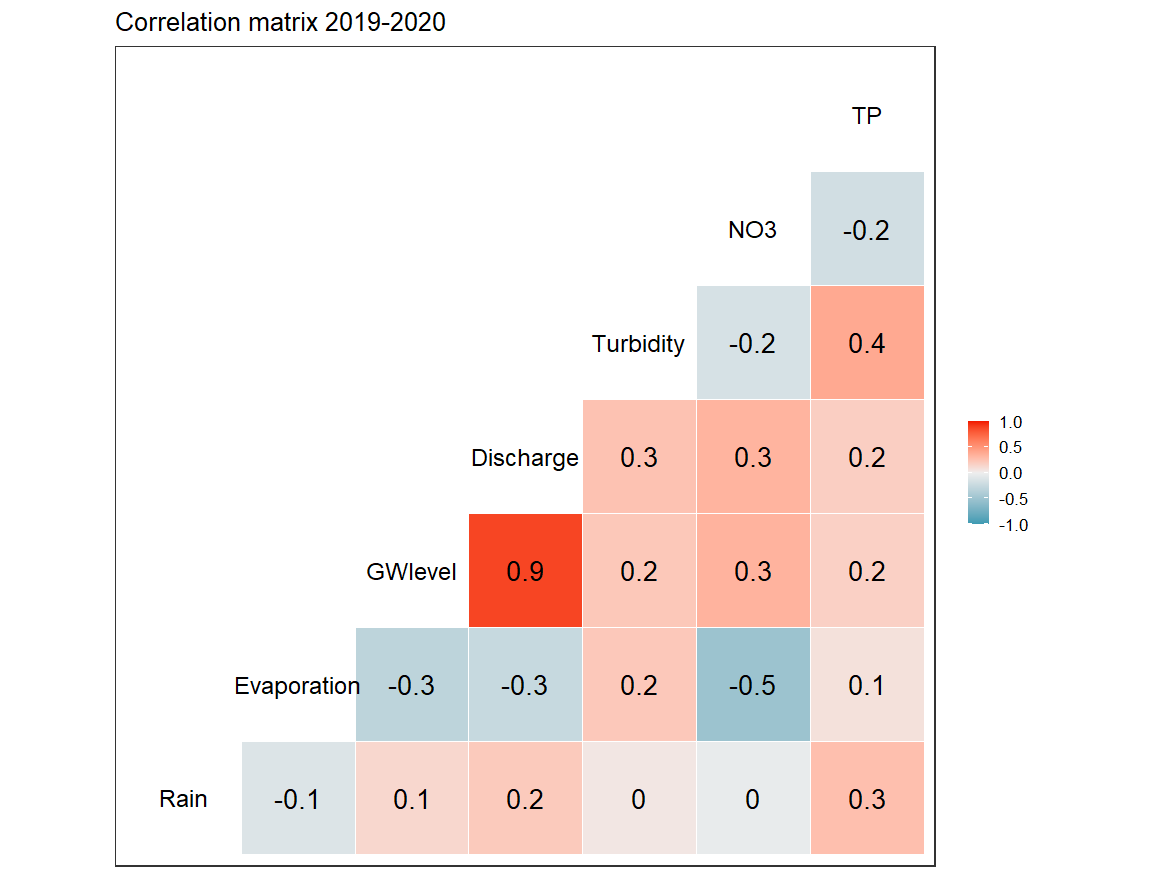 | 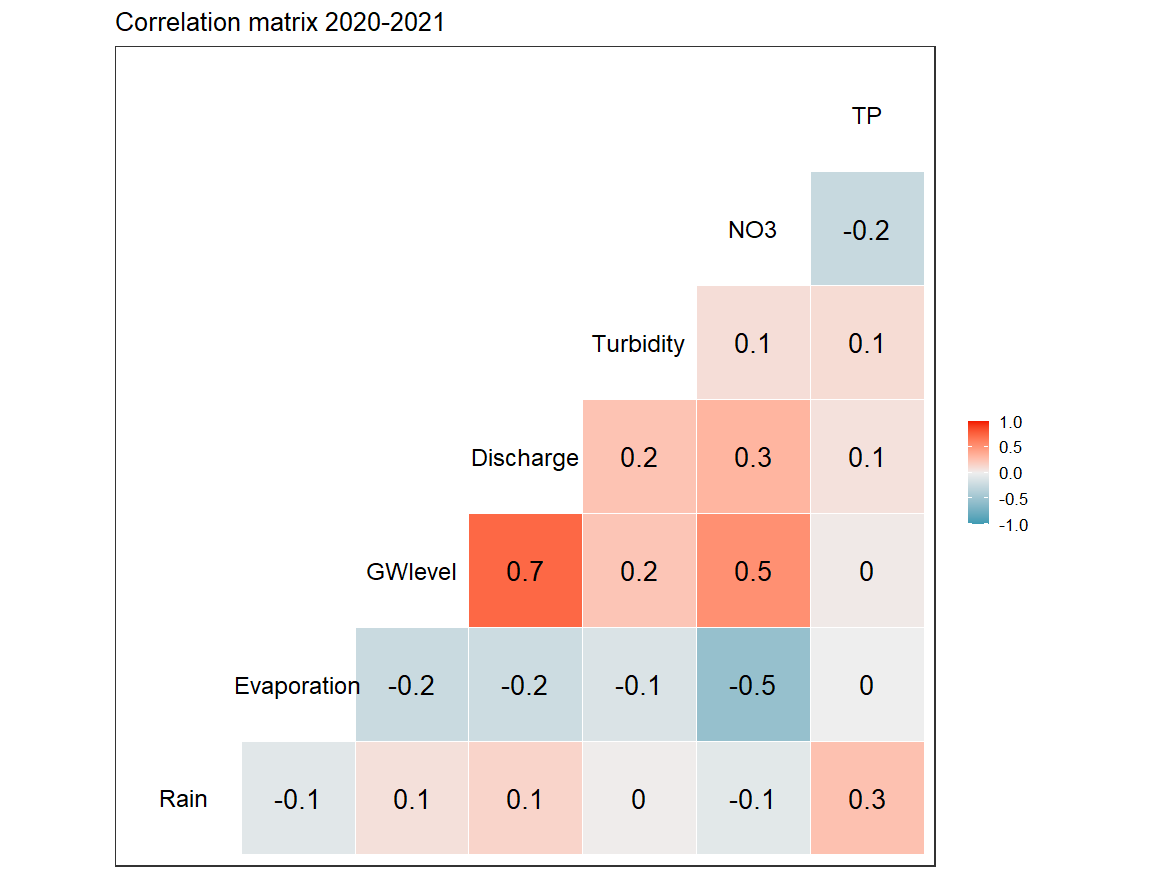 |

Figure S2 Matrix with the correlation coefficients of the measured data series in the four drainage seasons

Table S1 Statistical summary of the groundwater, turbidity, NO_3_, and TP data for all seasons

|  |  | All seasons | 2017-2018 | 2018-2019 | 2019-2020 | 2020-2021 |
| --- | --- | --- | --- | --- | --- | --- |
| Groundwater level (m) | Min* | -1.34 | -1.18 | -1.20 | -1.24 | -1.34 |
|  | 1Q | -1.18 | -1.06 | -1.04 | -1.04 | -0.907 |
|  | Median | -1.23 | -0.994 | -0.935 | -0.854 | -0.754 |
|  | Mean | -1.28 | -0.989 | -0.916 | -0.858 | -0.769 |
|  | 3Q | -0.898 | -0.917 | -0.838 | -0.726 | -0.630 |
|  | 95 perc | -0.261 | -0.630 | -0.417 | -0.301 | -0.261 |
| Turbidity (NTU) | Min | 0.21 | 0.93 | 0.21 | 0.40 | 1.04 |
|  | 1Q | 1.51 | 1.47 | 1.30 | 1.04 | 2.78 |
|  | Median | 2.59 | 1.82 | 2.03 | 2.19 | 3.51 |
|  | Mean | 9.40 | 2.06 | 14.1 | 4.89 | 13.4 |
|  | 3Q | 4.15 | 2.29 | 2.88 | 4.84 | 5.50 |
|  | 95 perc | 23.1 | 7.12 | 5.26 | 19.4 | 52.4 |
| NO_3_ (mg/L) | Min | 0.10 | 0.601 | 3.98 | 3.73 | 0.10 |
|  | 1Q | 6.81 | 3.23 | 8.46 | 9.73 | 6.29 |
|  | Median | 9.32 | 5.57 | 9.78 | 11.1 | 8.23 |
|  | Mean | 9.52 | 4.91 | 9.97 | 12.7 | 7.77 |
|  | 3Q | 11.18 | 6.49 | 11.7 | 15.0 | 10.1 |
|  | 95 perc | 20.81 | 3.46 | 13.63 | 23.0 | 11.37 |
| TP (mg/L) | Min | 0.005 | 0.005 | 0.007 | 0.016 | 0.023 |
|  | 1Q | 0.030 | 0.008 | 0.020 | 0.029 | 0.047 |
|  | Median | 0.040 | 0.005 | 0.026 | 0.036 | 0.059 |
|  | Mean | 0.050 | 0.010 | 0.035 | 0.045 | 0.077 |
|  | 3Q | 0.060 | 0.010 | 0.038 | 0.050 | 0.084 |
|  | 95 perc | 0.120 | 0.017 | 0.077 | 0.091 | 0.182 |

***Below this level there was no water flow discharge**

Table S2 Results of the different NO_3_ models, showing R^2^, MAE, RMSE, and computation times

|  |  |  | All seasons (2017-2021) | 2017-2018 | 2018-2019 | 2019-2020 | 2020-2021 |
| --- | --- | --- | --- | --- | --- | --- | --- |
| NO3 | Random Forest* | R^2^ | 0,996/0,995/0,995 | 0,998/0,998/0,998 | 0,998/0,998/0,998 | 0,995/0,994/0,994 | 0,998/,998/0,998 |
|  |  | MAE | 0,112/0,113/0,113 | 0,027/0,026/0,027 | 0,043/0,040/0,041 | 0,134/0,140/0,148 | 0,046/0,047/0,047 |
|  |  | RMSE | 0,311/0,322/0,328 | 0,039/0,036/0,040 | 0,086/0,074/0,086 | 0,375/0,357/0,415 | 0,077/0,080/0,080 |
|  |  | Time(s) | 118/102/100 | 0,52/0,54/0,48 | 5,45/6,10/5,66 | 22/22/22 | 21/23/22 |
|  | K-Nearest Neighbor | R^2^ | 0,735/0,735/0,721 | 0,737/0,753/0,712 | 0,998/0,998/0,998 | 0,821/0,812/0,812 | 0,846/0,848/0,846 |
|  |  | MAE | 1,21/1,20/1,23 | 0,638/0,615/0,709 | 0,018/0,017/0,017 | 0,900/0,925/0,926 | 0,567/0,565/0,561 |
|  |  | RMSE | 2,71/2,72/2,79 | 1,14/1,11/1,22 | 0,049/0,046/0,049 | 2,37/2,42/2,42 | 1,20/1,199/1,197 |
|  |  | Time(s) | 4151/2458/2161 | 8,38/7,52/8,33 | 6,69/6,18/6,81 | 84/73/97 | 128/103/93 |
|  | M5 rules | R^2^ | 0,941/0,877/0,968 | 0,996/0,996/0,996 | 0,988/0,984/0,984 | 0,964/0,964/0,924 | 0,988/0,982/0,980 |
|  |  | MAE | 0,454/0,503/0,368 | 0,071/0,074/0,070 | 0,158/0,156/0,156 | 0,471/0,471/0,503 | 0,179/0,193/0,166 |
|  |  | RMSE | 1,13/1,66/0,826 | 0,109/0,116/0,104 | 0,270/0,298/0,298 | 1,02/1,02/1,49 | 0,328/0,389/0,418 |
|  |  | Time(s) | 1494/1361/2143 | 12,97/14,09/12,03 | 34/26/30 | 137/124/119 | 282/258/287 |
|  | Multivariable Linear Regression | R^2^ | 0,389/0,386/0,388 | 0,974/0,843/0,841 | 0,612/0,621/0,621 | 0,322/0,329/0,327 | 0,522/0,518/0,519 |
|  |  | MAE | 2,48/2,49/2,48 | 0,583/0,585/0,590 | 1,10/1,11/1,10 | 3,59/3,55/3,57 | 1,54/1,55/1,55 |
|  |  | RMSE | 3,58/3,58/3,58 | 0,731/0,736/0,740 | 1,48/1,46/1,46 | 4,41/4,36/4,38 | 2,00/2,02/2,02 |
|  |  | Time(s) | 0,3/0,5/0,3 | 0,04/0,03/0,03 | 0,03/0,03/0,02 | 0,06/0,07/0,06 | 0,06/0,06/0,06 |
|  | Sequential minimal optimization | R^2^ | 0,386/0,379/0,384 | 0,838/0,848/0,848 | 0,604/0,615/0,612 | 0,293/0,300/0,300 | 0,497/0,497/0,493 |
|  |  | MAE | 2,39/2,40/2,40 | 0,565/0,572/0,572 | 1,0885/1,0902/1,0843 | 3,2899/3,2433/3,253 | 1,4976/1,4976/1,5094 |
|  |  | RMSE | 3,6367/3,6376/3,6362 | 0,768/0,783/0,783 | 1,49/1,47/1,48 | 4,78/4,72/4,73 | 2,06/2,06/2,08 |
|  |  | Time(s) | 154983/166755/173023 | 1320/1479/1728 | 1834/2020/1880 | 15876/19126/20921 | 18976/14022/18456 |
|  | Zero Rules | R^2^ | 0/0/0 | 0/0/0 | 0/0/0 | 0/0/0 | 0/0/0 |
|  |  | MAE | 3,25/3,24/3,25 | 1,64/1,65/1,64 | 1,92/1,94/1,93 | 4,25/4,24/4,24 | 2,25/2,27/2,26 |
|  |  | RMSE | 4,58/4,57/4,58 | 1,85/1,86/1,86 | 2,37/2,38/2,37 | 5,36/5,33/5,34 | 2,89/2,92/2,91 |
|  |  | Time(s) | 0,2/0,3/0,2 | 0,01/0,01/0,01 | 0,01/0,01/0,01 | 0,01/0,01/0,01 | 0,01/0,01/0,01 |
|  | Artificial Neural Networks | R^2^ | 0,663/0,667/0,669 | 0,978/0,980/0,980 | 0,880/0,882/0,880 | 0,672/0,664/0,664 | 0,870/0,863/0,863 |
|  |  | MAE | 1,87/2,28/2,28 | 0,205/0,201/0,201 | 0,603/0,879/0,603 | 2,16/2,10/2,10 | 0,896/0,812/0,812 |
|  |  | RMSE | 2,87/2,96/2,96 | 0,285/0,269/0,269 | 0,857/1,12/0,857 | 3,08/3,10/3,10 | 1,16/1,08/1,08 |
|  |  | Time (s) | 97/103/111 | 10/11/12 | 16/12/12 | 37/32/29 | 42/37/48 |

Table S3 Results of the different TP models, showing R^2^, MAE, RMSE, and computation times

|  |  |  | All seasons (2017-2021) | 2017-2018 | 2018-2019 | 2019-2020 | 2020-2021 |
| --- | --- | --- | --- | --- | --- | --- | --- |
| TP | Random Forest* | R^2^ | 0,963/0,958/0,958 | 0,960/0,975/0,969 | 0,934/0,934/0,934 | 0,927/0,917/0,917 | 0,972/0,967/0,967 |
|  |  | MAE | 0,002/0,003/0,003 | 0,001/0,001/0,001 | 0,002/0,002/0,002 | 0,002/0,002/0,002 | 0,003/0,003/0,003 |
|  |  | RMSE | 0,010/0,010/0,010 | 0,001/0,001/0,001 | 0,007/0,007/0,007 | 0,010/0,010/0,010 | 0,010/0,011/0,011 |
|  |  | Time(s) | 134/112/131 | 3,13/3,04/3,05 | 11,3/7,4/8,9 | 23/22/25 | 21/15/15 |
|  | K-Nearest Neighbor | R^2^ | 0,836/0,817/0,877 | 0,946/0,966/0,962 | 0,874/0,941/0,876 | 0,589/0,532/0,584 | 0,945/0,958/0,948 |
|  |  | MAE | 0,002/0,003/0,002 | 0,001/0,001/0,001 | 0,001/0,001/0,001 | 0,004/0,005/0,004 | 0,002/0,002/0,002 |
|  |  | RMSE | 0,020/0,021/0,017 | 0,002/0,001/0,001 | 0,011/0,007/0,010 | 0,028/0,032/0,027 | 0,014/0,012/0,014 |
|  |  | Time(s) | 1598/1455/1590 | 1,92/1,90/1,77 | 11,3/6,5/6,4 | 56/51/49 | 31/32/37 |
|  | M5 rules | R^2^ | 0,808/0,815/0,815 | 0,901/0,858/0,858 | 0,798/0,798/0,799 | 0,561/0,701/0,701 | 0,828/0,880/0,880 |
|  |  | MAE | 0,008/0,008/0,008 | 0,001/0,001/0,001 | 0,005/0,005/0,005 | 0,01/0,007/0,007 | 0,010/0,009/0,009 |
|  |  | RMSE | 0,021/0,020/0,020 | 0,002/0,002/0,002 | 0,013/0,013/0,012 | 0,024/0,019/0,019 | 0,025/0,021/0,021 |
|  |  | Time(s) | 1351/1151/1047 | 22/20/24 | 62/61/56 | 125/111/80 | 171/196/157 |
|  | Multivariable Linear Regression | R^2^ | 0,211/0,199/0,213 | 0,548/0,529/0,524 | 0,523/0,542/0,534 | 0,212/0,23/0,219 | 0,158/0,168/0,163 |
|  |  | MAE | 0,023/0,023/0,023 | 0,002/0,002/0,002 | 0,010/0,010/0,010 | 0,015/0,015/0,015 | 0,032/0,033/0,032 |
|  |  | RMSE | 0,041/0,042/0,042 | 0,004/0,005/0,004 | 0,020/0,019/0,019 | 0,031/0,031/0,030 | 0,053/0,055/0,054 |
|  |  | Time(s) | 1,6/0,5/0,5 | 0,01/0,01/0,01 | 0,05/0,06/0,06 | 0,06/0,05/0,06 | 0,06/0,04/0,04 |
|  | Sequential minimal optimization regression | R^2^ | 0,190/0,180/0,193 | 0,533/0,514/0,503 | 0,237/0,246/0,246 | 0,156/0,170/0,171 | 0,123/0,120/0,112 |
|  |  | MAE | 0,020/0,020/0,020 | 0,002/0,002/0,002 | 0,009/0,008/0,008 | 0,013/0,013/0,012 | 0,028/0,028/0,028 |
|  |  | RMSE | 0,042/0,043/0,043 | 0,005/0,005/0,004 | 0,030/0,029/0,030 | 0,033/0,034/0,032 | 0,056/0,058/0,059 |
|  |  | Time(s) | 60008/55637/51993 | 191/210/207 | 1098/1019/1018 | 7193/7126/4746 | 3559/5722/6457 |
|  | Zero Rules | R^2^ | 0/0/0 | 0/0/0 | 0/0/0 | 0/0/0 | 0/0/0 |
|  |  | MAE | 0,027/0,028/0,028 | 0,003/0,003/0,003 | 0,018/0,017/0,017 | 0,018/0,018/0,018 | 0,035/0,036/0,036 |
|  |  | RMSE | 0,046/0,047/0,047 | 0,007/0,007/0,006 | 0,029/0,027/0,028 | 0,035/0,036/0,034 | 0,058/0,060/0,060 |
|  |  | Time(s) | 0,8/0,6/1,5 | 0/0/0 | 0/0/0 | 0/0/0 | 0/0/0 |
|  | Artificial Neural Networks | R^2^ | 0,489/0,534/0,534 | 0,753/0,516/0,516 | 0,702/0,699/0,724 | 0,438/0,524/0,524 | 0,510/0,557/0,557 |
|  |  | MAE | 0,018/0,016/0,016 | 0,002/0,002/0,002 | 0,020/0,008/0,010 | 0,0124/0,0111/0,0111 | 0,0236/0,022/0,0226 |
|  |  | RMSE | 0,034/0,033/0,033 | 0,003/0,004/0,004 | 0,024/0,015/0,016 | 0,029/0,024/0,024 | 0,044/0,040/0,040 |
|  |  | Time(s) | 70/68/75 | 8/7/9 | 12/12/12 | 35/28/30 | 22/23/26 |

*** the RF times include computing the variable importance**

| **2017-2018** | **2018-2019** |
| --- | --- |
| 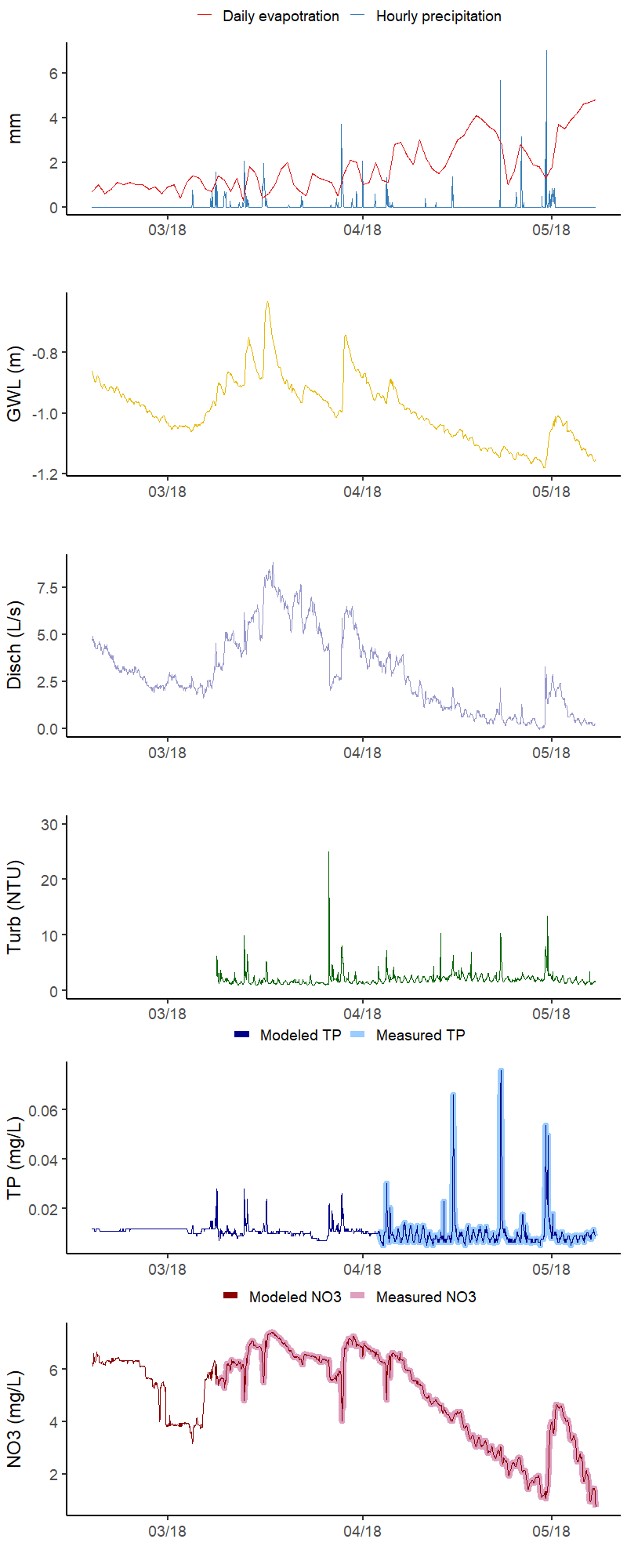 | 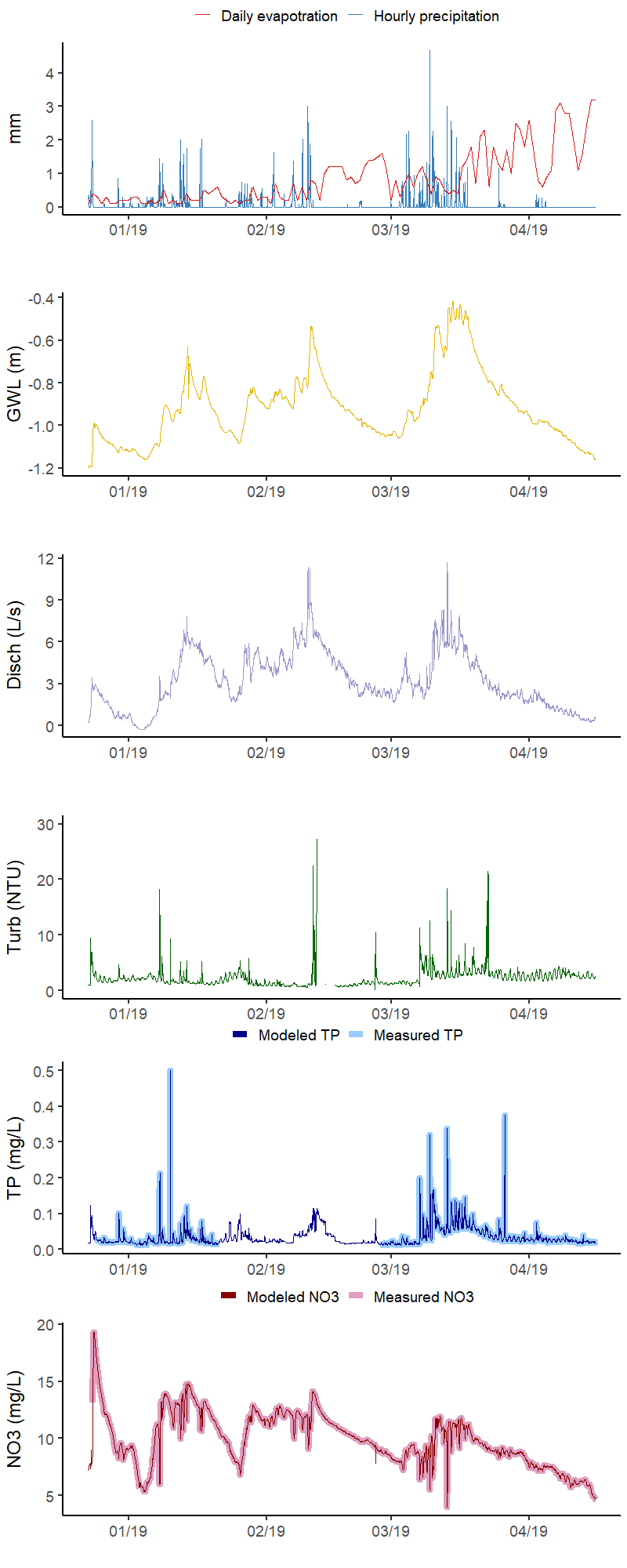 |

Figure S3 2017-2018 and 2018-2019 datasets with measured and modeled TP and NO3. The dash boxes show data gaps.

| 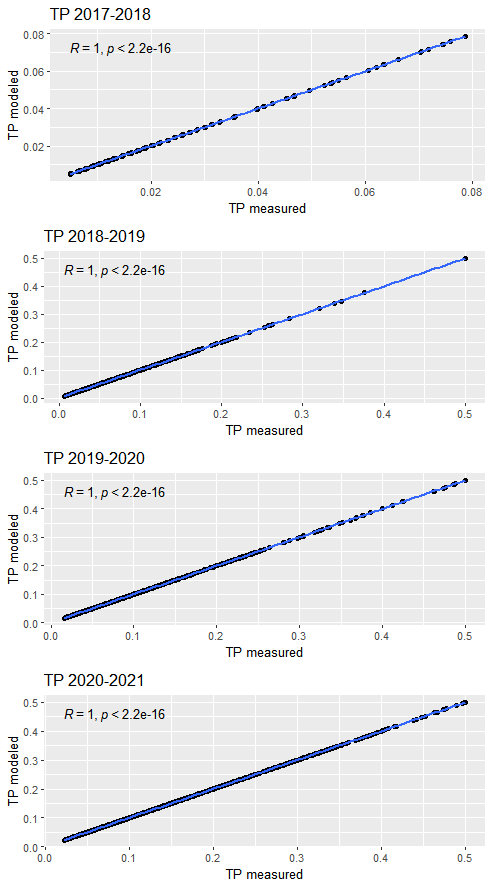 | 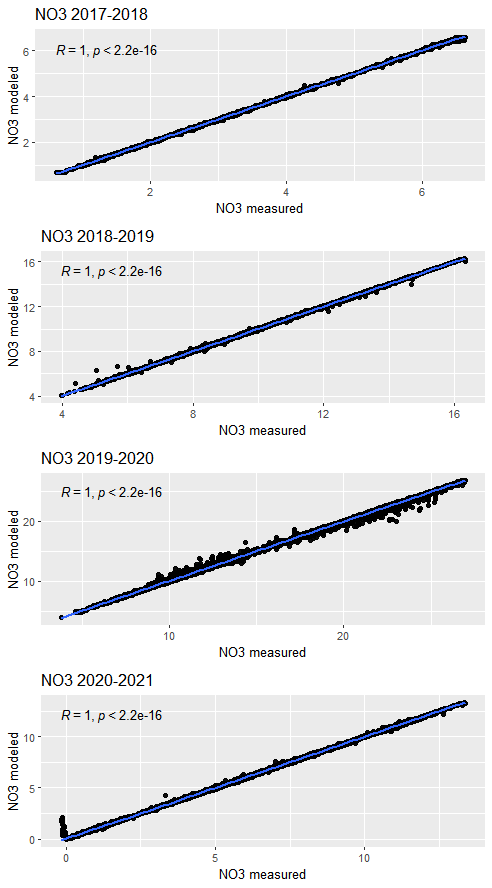 |
| --- | --- |

Figure S4 Correlation coefficients for TP and NO3 Random Forest models for each season

Seasonal loads vs time

| **TP export loads per season** | **NO_3_ export loads per season** |
| --- | --- |
| 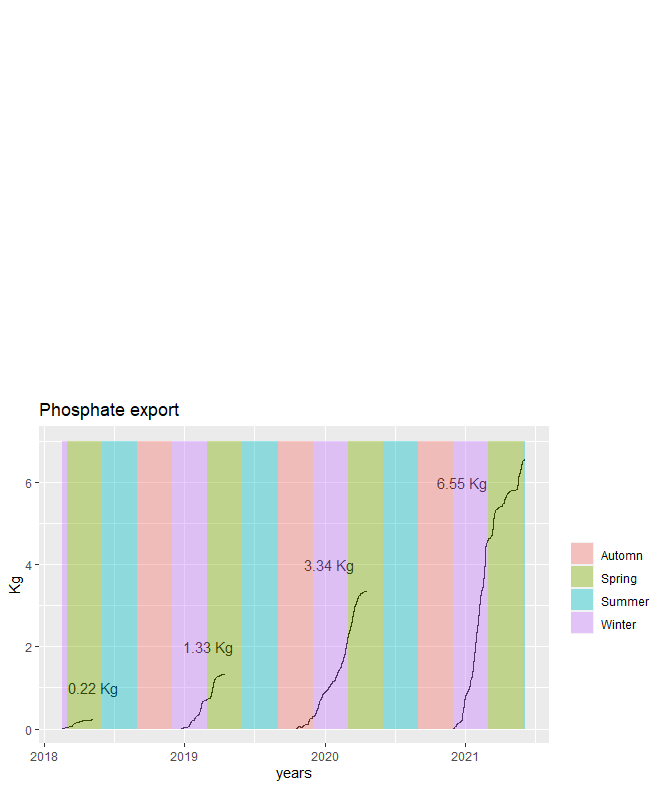 **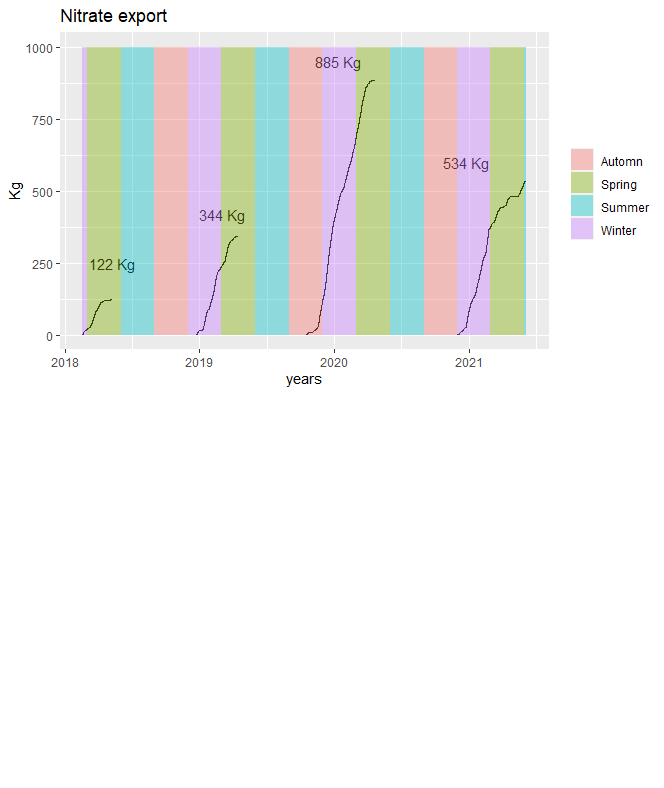** | |

Figure S5 NO3 and TP in the different drainage seasons: measured and modeled with Random Forest
